# *Mycobacterium tuberculosis* Cells Surviving in the Continued Presence of Bactericidal Concentrations of Rifampicin *in vitro* Develop Negatively Charged Thickened Capsular Outer Layer That Restricts Permeability to the Antibiotic

**DOI:** 10.3389/fmicb.2020.554795

**Published:** 2020-12-17

**Authors:** Jees Sebastian, Rashmi Ravindran Nair, Sharmada Swaminath, Parthasarathi Ajitkumar

**Affiliations:** Department of Microbiology and Cell Biology, Indian Institute of Science, Bengaluru, India

**Keywords:** *Mycobacterium tuberculosis*, rifampicin permeability, rifampicin surviving cells, thickened capsular outer layer, negatively charged polysaccharides

## Abstract

Majority of the cells in the bacterial populations exposed to lethal concentrations of antibiotics for prolonged duration succumbs to the antibiotics’ sterilizing activity. The remaining cells survive by diverse mechanisms that include reduced permeability of the antibiotics. However, in the cells surviving in the continued presence of lethal concentrations of antibiotics, it is not known whether any cell surface alterations occur that in turn may reduce permeability of the antibiotics. Here we report the presence of a highly negatively charged, hydrophilic, thickened capsular outer layer (TCOL) on a small proportion of the rifampicin surviving population (RSP) of *Mycobacterium tuberculosis* (*Mtb*) cells upon prolonged continuous exposure to bactericidal concentrations of rifampicin *in vitro*. The TCOL reduced the intracellular entry of 5-carboxyfluorescein-rifampicin (5-FAM-rifampicin), a fluorochrome-conjugated rifampicin permeability probe of negligible bacteriocidal activity but comparable properties. Gentle mechanical removal of the TCOL enabled significant increase in the 5-FAM-rifampicin permeability. Zeta potential measurements of the cells’ surface charge and hexadecane assay for cell surface hydrophobicity showed that the TCOL imparted high negative charge and polar nature to the cells’ surface. Flow cytometry using the MLP and RSP cells, stained with calcofluor white, which specifically binds glucose/mannose units in β (1 → 4) or β (1 → 3) linkages, revealed the presence of lower content of polysaccharides containing such residues in the TCOL. GC-MS analyses of the TCOL and the normal capsular outer layer (NCOL) of MLP cells showed elevated levels of α-D-glucopyranoside, mannose, arabinose, galactose, and their derivatives in the TCOL, indicating the presence of high content of polysaccharides with these residues. We hypothesize that the significantly high thickness and the elevated negative charge of the TCOL might have functioned as a physical barrier restricting the permeability of the relatively non-polar rifampicin. This might have reduced intracellular rifampicin concentration enabling the cells’ survival in the continued presence of high doses of rifampicin. In the context of our earlier report on the *de novo* emergence of rifampicin-resistant genetic mutants of *Mtb* from the population surviving under lethal doses of the antibiotic, the present findings attain clinical significance if a subpopulation of the tubercle bacilli in tuberculosis patients possesses TCOL.

## Introduction

*Mycobacterium tuberculosis* (*Mtb*), the pathogenic bacterium that causes tuberculosis, possesses remarkable structural and functional features that enable their survival under various stress conditions, including lethal concentrations of antibiotics. One of such features, the uniquely structured cell wall (Daffe, 2015; Jankute et al., 2015), is believed to contribute to the refractoriness of the bacilli toward many antibiotics. The physiological significance of the changes in the cell wall of mycobacteria residing in macrophages (Larrouy-Maumus et al., 2016), under nutritional stress conditions (Nyka, 1974; Bacon et al., 2014), hypoxia (Cunningham and Spreadbury, 1998) and in pathogenicity (Daffé and Etienne, 1999) has been previously studied. The complex structure of the cell wall of *Mtb* is also believed to restrict its permeability toward several host-derived antimicrobial biomolecules, thereby enabling the survival of the bacilli in its diverse habitats (Lederer et al., 1975; Jarlier and Nikaido, 1994; Cook et al., 2009; Nguyen, 2016). However, it is not known whether the antibiotic surviving *Mtb* subpopulations develop any specific structural features of the cell envelope that may ensure sub-lethal intracellular concentration of antibiotics despite being continuously exposed to high concentrations of the antibiotics.

One of the mechanisms for ensuring low levels of intracellular concentrations of antibiotics in actively growing bacteria is the decreased permeability of antibiotics into the cells. The reasons for the reduced permeability are cell-wall thickening against rifampicin in *Neisseria meningitidis* (Abadi et al., 1996), vancomycin in *Staphylococcus aureus* (Cui et al., 2003), and adaptive resistance to amikacin in clinical isolates of methicillin-resistant *Staphylococcus aureus* (MRSA) (Yuan et al., 2013). Increased thickening of cell wall resulting in reduced intracellular antibiotic concentration has been found to be caused also by Rv0071/74 gene fusion due to RD105 region deletion in some clinical strains of *Mtb* (Qin et al., 2019). Restricted membrane permeability has been found to contribute to rifampicin resistance in actively growing mycobacteria as well (Hui et al., 1977).

*Mtb* strains containing high poly (P) levels due to exopolyphosphatase (ppx2) gene deficiency have been found to have increased cell-wall thickness and consequent reduced drug permeability (Chuang et al., 2015). Further, oxygen reduction in latent mycobacteria has been found to cause cell wall thickening (Velayati et al., 2011). We have recently reported thickening of capsular outer layer, caused by the formation of specific types of polysaccharides in unusually high abundance, in the hypoxic non-replicating persistent *Mtb* cells, which restricted rifampicin entry thereby enabling survival (Jakkala and Ajitkumar, 2019). Nutrient starved non-replicating *Mtb* cells also have shown reduced antibiotic entry (Sarathy et al., 2013). Besides cell-wall thickening, in many cases, the capsular polysaccharides have been associated with restricting antibiotic penetration into the cells, resulting in drug resistance (Slack and Nichols, 1982).

We recently reported that a small proportion of *Mtb* and *Mycobacterium smegmatis* (*Msm*) mid-log phase (MLP) cultures exposed to bactericidal concentrations of rifampicin and moxifloxacin survive for durations as long as 20 days and 96 h and more for *Mtb* and *Msm*, respectively, despite the continued presence of lethal concentrations of the antibiotics (Sebastian et al., 2017; Swaminath et al., 2020a, b). However, it is not known whether any cell surface alterations occurred in these antibiotic surviving cells in the continued presence of lethal concentrations of the antibiotics. Further, if such alterations do occur, whether they have any role in ensuring sub-lethal intracellular concentrations of rifampicin in the rifampicin surviving cells *in vitro*. Therefore, here we analyzed the ultrastructural features of the surface of *Mtb* cells that might have enabled restricted rifampicin permeability and survival *in vitro*.

## Materials and Methods

### Generation of Rifampicin Surviving Population of *Mtb in vitro*

*Mycobacterium tuberculosis* H_37_R_a_ (*Mtb*), (JALMA Institute of Leprosy and Other Mycobacterial Diseases, India) was cultured in biological triplicates in Middlebrook 7H9 medium (Middlebrook et al., 1947) containing glycerol (0.2%), Tween 80 (0.05%) and ADS (albumin, dextrose, sodium chloride) (10%), at 37°C in bacteriological incubator with shaking at 170 rpm. The mid-log phase (MLP, 0.6 at OD_600 nm_) bacterial culture was treated with 10× minimum bactericidal concentration (MBC, 1 μg/ml) of rifampicin (Sigma) and incubated for 24 days. Once in every 24 h, aliquots were withdrawn from the cultures and plated on rifampicin-free Middlebrook 7H10 agar plates with 10% ADS, as described. The plates were incubated at 5% CO_2_ and 37°C in CO_2_ incubator for 3–4 weeks. The colony forming units (CFUs) were plotted against exposure time to get rifampicin susceptibility profile, from which the antibiotic surviving phase was identified, as described (Sebastian et al., 2017).

### Bioassay for 5-FAM-Rifampicin

*Staphylococcus aureus* (ATCC 25923), which is a rifampicin-sensitive strain, was used for the bioassay of 5-FAM-rifampicin, as described (Dickinson et al., 1974; Sebastian et al., 2017). In brief, the stock solutions (2 mg/ml) of rifampicin (sigma) and 5-FAM-rifampicin were made in DMSO. Different dilutions of these solutions were used for the bioassay, as described (Dickinson et al., 1974; Sebastian et al., 2017). Fifty microliters of the *S. aureus* glycerol stock were mixed with 100 ml LB agar (warm to the touch) and poured to make LB agar plates embedded with the bacilli. A stainless-steel puncture of 0.5 cm diameter was used to make diffusion wells in the agar. Fifty microliters aliquots of solutions containing increasing concentrations of rifampicin or 5-FAM-rifampicin were added into the well. The plates were incubated overnight at 37°C in a bacteriological incubator. The zone of inhibition (ZOI) of the bacillary growth was determined by measuring the diameter of the zone using Vernier Caliper. The ZOI values obtained from the rifampicin plates were used for plotting the standard graph for rifampicin. From this standard graph, the ZOI of 5-FAM-rifampicin was determined, from which the relative bioactivity of 5-FAM-rifampicin was estimated.

### Fluorescence Microscopy

The MLP cells and the cells from the rifampicin surviving population were used for flow cytometry experiments. The cells were resuspended in 7H9 medium (100 μl). The rifampicin permeability probe, 5-FAM-rifampicin (1.5 μg/ml), was added into the cell suspensions, which were then incubated in bacteriological incubator shaker for 1 h at 37°C. To discriminate dead cells from live cells, 1:1000 dilution of propidium iodide (PI) and incubated in multi-well slides, coated with poly-L-lysine, for 20 min in the dark. The cells were washed once with PBS and mounted with glycerol and observed under 100× objective using fluorescence microscope (Zeiss AxioVision). For removing the capsular outer layer, bead-beating was performed. For this, the cells in 20 ml culture were incubated with sterile glass beads (4 mm diameter; 10 g) at 37°C for 15 min at 50 rpm, as described (Ortalo-Magne et al., 1995), with minor modifications. Subsequently, 5-FAM-rifampicin was added and processed as described below.

### 5-FAM-Rifampicin Permeability Assay Using Flow Cytometry

The cells from the MLP cultures and from the rifampicin surviving population were used for the 5-FAM-rifampicin permeability assay. Cell suspensions (20 ml) were exposed to 1.5 μg/ml (final concentration) of 5-FAM-rifampicin, which is equivalent to 10× MBC rifampicin, by incubating them in a bacteriological shaker incubator at 37°C. For removing the outer capsular layer, the MLP and the cells from the rifampicin surviving population were gently beaten with 4 mm glass beads for 5 min at 50 rpm in a shaker incubator, as described (Ortalo-Magne et al., 1995), with minor modifications. At every 15 min interval, aliquots of the cultures were collected and washed once with ice-cold 7H9 broth and processed for flow cytometry analysis in BD FACSVerse system. The median autofluorescence of the control samples were kept at 10^2^ and the relative fluorescence of the stained samples were calculated. The statistical significance of the 60 min median fluorescence between the samples was estimated using two-tailed paired *t*-test.

In each sample, the whole population (all events) was gated to obtain the P1 population. The exact number of cells in the P1 population showing higher 5-FAM fluorescence than the remaining cells in the P1 population was obtained using the polygonal P2 gate. The polygonal P2 gate helped to exclude the cells with lower fluorescence in the P1 population. Further, the P2 population was obtained by placing the polygonal P2 gate exactly where the P1 population ended. This helped in getting the actual number of the cells in the P1 population showing higher 5-FAM median fluorescence than the remaining cells in the P1 population. The P2 cells represented the cells that gained 5-FAM fluorescence during the 60 min exposure.

A standard graph was constructed for the calculation of the extent of 5-FAM-rifampicin permeability. For this, the *Mtb* MLP cells were incubated with increasing concentrations of 5-FAM-rifampicin for 1 h at 37°C. Subsequent to incubation, the cells were washed once with ice-cold Middlebrook 7H9 broth and processed for flow cytometry analysis using 488 nm laser with 527/32 filter. The normalized median fluorescence values of 5-FAM-rifampicin were plotted against the concentration to get the standard graph. The cells from the 12th day of the treatment were taken as the cells from the rifampicin surviving population (Sebastian et al., 2017). The cells from the rifampicin surviving population were incubated with 5-FAM-rifampicin, at a concentration that was equivalent of 10x MBC rifampicin. The fluorescence values from six independent samples of the cells from the rifampicin surviving population (10,000 gated cells) were normalized with autofluorescence values and plotted. The relative concentrations of 5-FAM-rifampicin in the cells from the rifampicin surviving population were calculated from the standard graph.

### Hexadecane Hydrophobicity Assay for Bacterial Cells

The hydrophobicity assay was performed by using a previously described method (Rosenberg et al., 1980; Stokes et al., 2004), with modifications. The MLP cells and the cells from the rifampicin surviving population (from 100 ml culture each) were suspended in filter-sterilized PUM buffer (50 mM KH_2_PO_4_, 100 mM K_2_HPO_4_.3H_2_O, 1 mM MgSO_4_.7H_2_O, and 33.3 mM urea, pH 7.1) prepared in double-distilled autoclaved water, in siliconised borosilicate tubes to get an OD_600 nm_ of ∼0.7 (>10^6^ cells/ml). One volume of PUM buffer containing cells was phase-extracted with three volumes of *n*-hexadecane by vortexing for 8–10 s and left at 25°C for 15 min. Using siliconised tips, the cells retained in the aqueous phase were collected, mildly sonicated, serially diluted and plated over 7H10 agar plates. The percentage of cells with the hydrophilic outer layer was calculated from the difference in the CFU of the cells in the aqueous phase, before and after phase extraction.

### Determination of Zeta Potential of *Mtb* Cells

The cells from the mid-log phase, killing phase, phase of the rifampicin surviving population and regrowth phase were collected and washed with Middlebrook 7H9 broth. The zeta potential of the cells was measured in Middlebrook 7H9 broth (unless otherwise mentioned) by using the zeta sizer (Nano-ZS90) instrument, as described (Wilson et al., 2001; Ayala-Torres et al., 2014). The isoelectric point (pI) of the cells were determined by resuspending the cells in buffer solutions of varying pH values of 2 to 10. The buffers of different pH values were prepared in PPMS (20 mM of KH_2_PO_4_, 1.5 mM of MgSO_4_.7H_2_O, and 40 mM of K_2_HPO_4_ per liter of Milli Q water) and the zeta potential was measured.

### Transmission Electron Microscopy

The *Mtb* cells from the MLP and rifampicin surviving population were used for transmission electron microscopy (TEM) using a method previously reported (Takade et al., 1983), with minor modifications standardized in our laboratory (Vijay et al., 2012). All the reagents used for sample preparation were electron microscopy grade from Sigma-Aldrich, unless mentioned. All the reagents for TEM were prepared fresh in the required quantity in double-distilled water, wherever required. In short, the cells were first prefixed with osmium tetroxide (1% w/v solution in water) in 0.15 M sodium cacodylate buffer of pH 7.2 for 1 h at 25°C and washed again with the same buffer. The cells were then fixed with glutaraldehyde and tannic acid (2% v/v each in 0.15 M sodium cacodylate buffer) for 2 h at 25°C. The cells, after fixation, were washed once again with the sodium cacodylate buffer and further fixed in osmium tetroxide for 14 h at 4°C. The fixed cells were then dehydrated using a series of washes with 25, 50, 75, and 95% ethanol. The cell pellet was then infiltrated with 50% LR white resin (prepared in 50% ethanol) for 24 h at 4°C. The cell pellets were made into blocks in gelatine capsules using 100% LR white resin and incubated at 65°C for 2–3 days in a dry bath for solidification. The blocks were then sectioned into 80–100 nm thickness using ultramicrotome. The sections were then placed on electron microscopy grade copper grids (150 mesh, Sigma), stained sequentially with 0.5% uranyl acetate and 0.04% lead citrate. The air-dried samples were then examined using electron microscopy (JEOL-100 CXII) at 100 kV. A total of 126 cells from rifampicin surviving population and 109 cells from MLP (tannic acid negative) were collected from biological triplicate cultures and the images of 30 cells each were measured from multiple sides of each of the 30 cells to determine the average thickness of the capsular outer layer.

### Flow Cytometry Analysis of Calcofluor White Stained Cells

Aliquots (500 μl) of the cultures, before and after bead-beating, were stained with 1:1,000 dilution of calcofluor white (CFW; Disodium salt of 4, 4′-bis-[4-anilino-bis-diethyl amino-S-triazin2-ylamino]-2, 2′-stilbene-disulphonic acid; 0.1% solution, Sigma; Maeda and Ishida, 1967) for one hr at 37°C, as described (Wood, 1980; Trivedi et al., 2016). The cells were quickly washed once with Middlebrook 7H9 medium and used for flow cytometry analysis in BD FACSVerse flow cytometer using 405 nm laser with 448/45 filter. The median fluorescence values of the CFW-stained cell samples were determined by keeping 2-log_10_ value of autofluorescence. The flow cytometry data were analyzed using FACSuite software. The P1 population was obtained by gating the whole population (all events) in each sample. The polygonal P2 gate was used to analyze the exact number of the cells in the P1 population showing higher CFW fluorescence than the remaining cells in the P1 population (polygonal P2 gate helped to exclude the cells with lower fluorescence in the P1 population). Further, the P2 gate was placed exactly where the P1 population ended for gating the actual number of cells showing higher CFW median fluorescence than the remaining cells in the P1 population. The photomultiplier tube voltage settings were 208 (FSC), 333 (SSC). The calibration of the instrument was performed using FACSuite cytometer set up and tracking beads (CS&T, Becton Dickinson). Data were processed and analyzed as described above under 5-FAM-rifampicin permeability analysis. Paired *t*-test was used to calculate statistical significance.

### GC-MS Profiling of Polysaccharides in the Capsular Outer Layer

The GC-MS analyses of the OL components isolated from the MLP and the cells from the rifampicin surviving population were performed, as previously described (Ortalo-Magne et al., 1995). In brief, the cells were first washed once with 1× PBS. The washed cells were resuspended in 20 ml distilled water and incubated with 4 mm diameter sterile glass beads (10 g) in a shaker incubator at 50 rpm for 15 min at 25°C. The cell suspension was centrifuged at 12,000 × *g* for 10 min and the supernatant was collected, filtered (0.2 micron filter) and lyophilised. The sample was derivatised as per the protocol at the glycomics mass spectrometry facility at the Centre for Cellular and Molecular Platforms (C-CAMP), Bangalore. The GC-MS analysis was performed on the derivatised samples and the data was analyzed at the facility. The percentage “relative abundance” of each compound in the capsular outer layer of MLP and rifampicin surviving population was independently determined from the GC-MS data. By comparing these values for each compound, the fold difference was calculated. Being a relative quantitation, normalization for the values was not performed with any parameter.

## Results

### Experimental Rationale and Strategy

The experiments were designed to find out whether rifampicin permeability was altered in the *Mtb* cells surviving in the continued presence of lethal doses of rifampicin and, if the permeability was reduced, whether it involved any cell surface alterations at the ultrastructural and components’ levels. For this purpose, we exposed *Mtb* cells to 10× MBC rifampicin for 20 days, as described earlier, to prepare the rifampicin-surviving population (Sebastian et al., 2017), which was the experimental sample. The antibiotic surviving population might contain subpopulations of classical ‘persisters’ as termed by Bigger (1944) and defined recently (Balaban et al., 2019), persisters that grew and divided slowly, like the slow growing and dividing isoniazid persisters of *M. smegmatis* (*Msm*) (Wakamoto et al., 2013), classical antibiotic tolerant cells as defined (Brauner et al., 2016; Windels et al., 2019), and probably many other subpopulations of hitherto unknown phenotypes. Any, or all, of these subpopulations might show reduced rifampicin permeability and it will be difficult to isolate and identify them individually using defined tests as their proportions will be very low. Hence, we examined the permeability of rifampicin into the whole rifampicin surviving population, in comparison to that into the MLP population.

**FIGURE 1:**
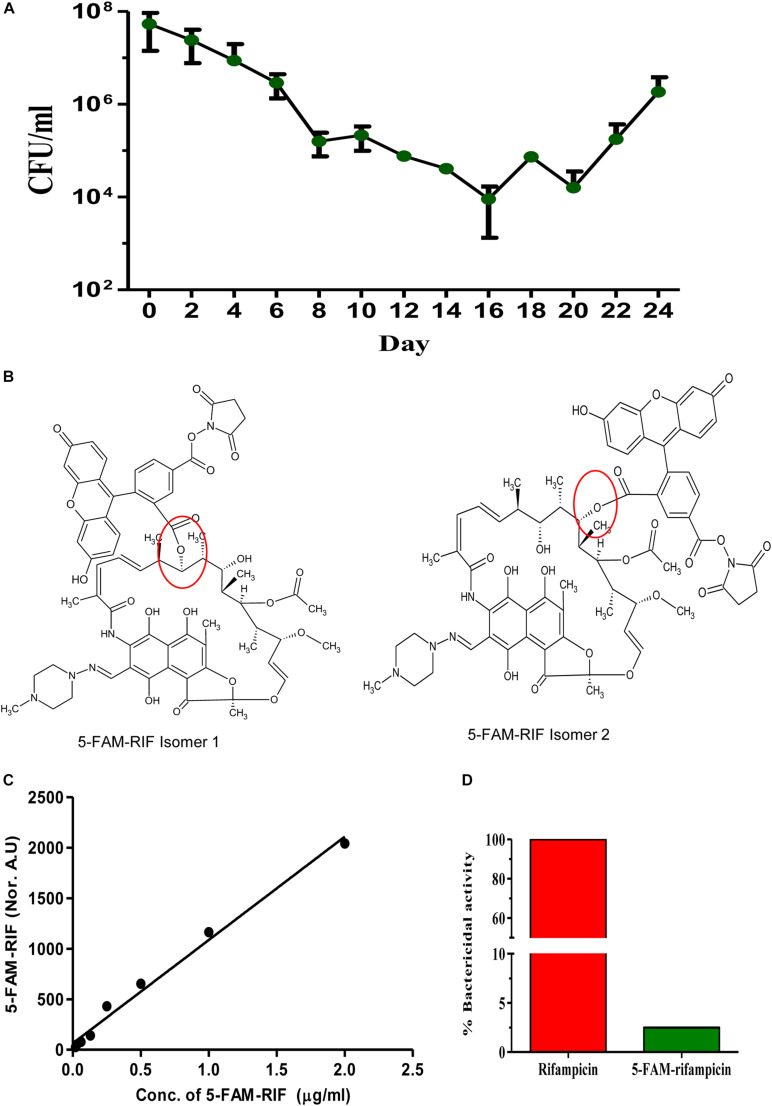
Triphasic response of *Mtb* culture to rifampicin and validation of 5-FAM-rifampicin as a permeability probe for rifampicin. **(A)** Triphasic response of *Mtb* culture to 10× MBC rifampicin. **(B)** The possible structural isomers of 5-FAM-rifampicin conjugates. The carboxyl group of the 5-FAM can form an ester bond with any one of the hydroxyl groups in the aliphatic ring of rifampicin, generating two possible isomers. Ester bond is shown in red circle. This structure of 5-FAM-rifampicin is a modified version of the independent structures of the rifampicin and 5-FAM shown in the Supplementary Figures S3A,B in Jakkala and Ajitkumar (2019). **(C)** Standard graph showing the 5-FAM-rifampicin permeability into MLP cells at two-fold increasing concentrations. **(D)** Bar graph showing the growth inhibition of *Staphylococcus aureus* by rifampicin and 5-FAM-rifampicin using agar diffusion assay.

We had earlier reported that *Mtb* MLP cells, when exposed to 10× MBC (1 μg/ml) of rifampicin for prolonged duration (20 days), consistently and reproducibly showed the profile of three sequential phases with respect to the CFUs. These were the killing phase with steep reduction in CFU (up to day 10 of the exposure), followed by a population showing only slight change in the CFUs (day 10 to day 15 of the exposure), which we called the rifampicin surviving population, which was followed by regrowth phase with steady rise in CFU (from day 15 onward) of rifampicin resisters (Sebastian et al., 2017). During the phase of rifampicin surviving population, we found rifampicin concentration still at ∼8–4× MBC (Sebastian et al., 2017). We found similar reproducible profile in the present study as well (Figure 1A). Therefore, we chose the day 12, the mid-point of the rifampicin-surviving phase, as the source of the cells for all the experiments.

The permeability of the rifampicin-surviving population was determined using a fluorophore-conjugated rifampicin, 5-carboxyfluorescein-rifampicin (5-FAM-rifampicin), before and after gentle mechanical removal of the extracellular capsular polysaccharide layer (ECPL) since it has been implicated in the permeability of antibiotics and other cellular properties of *Mtb* cells (Hui et al., 1977; Velayati et al., 2011; Sarathy et al., 2013). Having found significantly reduced permeability of the probe, we studied the biophysical properties of the ECPL, using hexadecane assay for hydrophobicity of the cell surface and zeta potential measurement to determine the surface charge, isoelectric point (pI) and thereby the polar/non-polar character of the surface of the cells. The biochemical nature of the ECPL of the cells from the rifampicin surviving population was further studied using fluorescence microscopy and flow cytometry with the cells stained with polysaccharide specific dyes before and after the removal of the ECPL. The ultrastructural features of the cells from the rifampicin surviving population were examined using transmission electron microscopy. Upon finding the ECPL on the MLP cells had become a thickened capsular outer layer (TCOL) on the surface of the cells in the rifampicin surviving population, its molecular composition was determined using GC-MS. The biophysical and biochemical properties of the TCOL were correlated with the reduced 5-FAM-rifampicin permeability of the cells in the rifampicin surviving population.

### Functional Validation of the 5-FAM-Rifampicin Permeability Probe

We examined the rifampicin permeability of the rifampicin surviving population using the fluorophore, 5-carboxyfluorescin (5-FAM), conjugated to rifampicin to get 5-FAM-rifampicin (Figure 1B). The presence of two aliphatic hydroxyl groups in rifampicin gave the possibility for the generation of two isomers upon the formation of either of the two ester bonds with 5-FAM to yield 5-FAM-rifampicin (Figure 1B). This preparation was used for the permeability assay. Rifampicin, a relatively non-polar molecule that is believed to passively diffuse into mycobacterial cells, was found to reach a steady state concentration of 154 ng/mg of mycobacterial cells in 2 min of exposure in an earlier study (Piddock et al., 2000). The small size and the non-polar nature of the 5-FAM were believed to maintain such features of rifampicin in 5-FAM-rifampicin. Thus, in view of these properties of the 5-FAM group, it was expected to be a fair candidate for conjugating with rifampicin as a fluorophore.

The concentration-dependent entry, when exposed to two-fold increase of concentrations of 5-FAM-rifampicin, into the actively growing *Mtb* MLP cells, as quantitated using flow cytometry, confirmed its high level of permeability into the cells (Figure 1C and Supplementary Figures 1A,B). Thus, the permeability of rifampicin into the actively growing *Mtb* MLP cells did not seem to have significantly affected by its conjugation with 5-FAM. Therefore, the fluorescence intensity of 5-FAM-rifampicin, as monitored using flow cytometry where only intact cells will be counted as events, could be considered as a measure of its permeability and accumulation in the bacterial cells. Thus, even if one considers the possibility of the probe getting non-specifically bound to dead cells, the flow cytometry measurements will not read them as events. These characteristics of the high level of permeability of 5-FAM-rifampicin into *Mtb* MLP cells validated its use as a permeability probe for rifampicin entry into the cells in the rifampicin surviving population, in comparison to its entry into MLP cells.

In addition, the 5-FAM-rifampicin possessed reduced bactericidal activity on *S. aureus*, and it was only 2.5% of the original bactericidal activity of rifampicin (Figure 1D and Supplementary Figures 1C,D). The minimum inhibitory concentration (MIC) of rifampicin was 0.05 μg/ml for both *Mtb* and *S. aureus* although *Mtb* was twice more sensitive to desacetyl rifampicin, a rifampicin metabolite found in the serum of tuberculosis patients (Maggi et al., 1968). Therefore, we presumed that 5-FAM-rifampicin also might elicit low bactericidal activity on *Mtb*, like on *S. aureus*. Moreover, the incubation of *Mtb* cells from the MLP and the rifampicin surviving population, with 5-FAM-rifampicin, was only for one hr, which is 1/24th of its generation time. Thus, the low bactericidal activity of 5-FAM-rifampicin on *Mtb*, as that on *S. aureus*, would considerably avoid cell death which otherwise would have interfered with the permeability assay on the cells from the rifampicin surviving population. For the biocidal reason, the use of radioactively labeled ^14^C/^3^H-rifampicin was avoided as they would still be natively structured as rifampicin, thereby inflicting lethality on the cells, thereby interfering with the experiments.

### Reduced Permeability of 5-FAM-Rifampicin Into Rifampicin Surviving Population

#### Qualitative Analysis of the Permeability Using Fluorescence Microscopy

First, the permeability of the cells of the rifampicin surviving population to 5-FAM-rifampicin was qualitatively studied using fluorescence microscopy. The cells in the rifampicin surviving population and MLP cells (as the positive control) were exposed to 1.5 μg/ml of 5-FAM-rifampicin for 1 h. The cells of the rifampicin surviving population showed low levels of fluorescence, as compared to the MLP cells, indicating that the permeability of 5-FAM-rifampicin might have been restricted in the cells of the rifampicin surviving population (Supplementary Figure 2A).

Antibiotic tolerance has been found to be due to altered membrane permeability in *N. meningitidis* (Abadi et al., 1996) and mycobacteria (Hui et al., 1977), and due to cell wall thickening in *S. aureus* (Cui et al., 2003). Since *Mtb* possesses characteristic capsular outer layer (Daffé and Etienne, 1999), which can be dissociated by gentle bead beating (Daffe and Laneelle, 2001), we wanted to find out whether removal of the capsular outer layer would enhance or restore 5-FAM-rifampicin permeability in the rifampicin surviving population, as compared to that in MLP. Consistent with this premise, gentle bead-beating of the cells of the rifampicin surviving population enhanced the 5-FAM fluorescence indicating increased permeability of 5-FAM-rifampicin into the cells (Supplementary Figure 2B, compare with Supplementary Figure 2A). However, bead-beating of the MLP cells did not make a difference in the 5-FAM-rifampicin fluorescence (Supplementary Figure 2B, compare with Supplementary Figure 2A). Thus, bead beating caused increased permeability of 5-FAM-rifampicin into the *Mtb* cells in the rifampicin surviving population but not in the MLP population. This difference suggested that: (i) the capsular outer layer of the cells in the rifampicin surviving population might be different from that in the MLP cells; (ii) the capsular outer layer of the *Mtb* cells in the rifampicin surviving population might be contributing to restricted 5-FAM-rifampicin permeability into those cells. These possibilities indicated that the natural capsular outer layer of the MLP cells, when exposed to lethal doses of rifampicin for prolonged duration, might have undergone alterations in the rifampicin surviving population that in turn might have restricted 5-FAM-rifampicin permeability.

#### Quantitative Analysis of the Rifampicin Permeability Using Flow Cytometry

**FIGURE 2:**
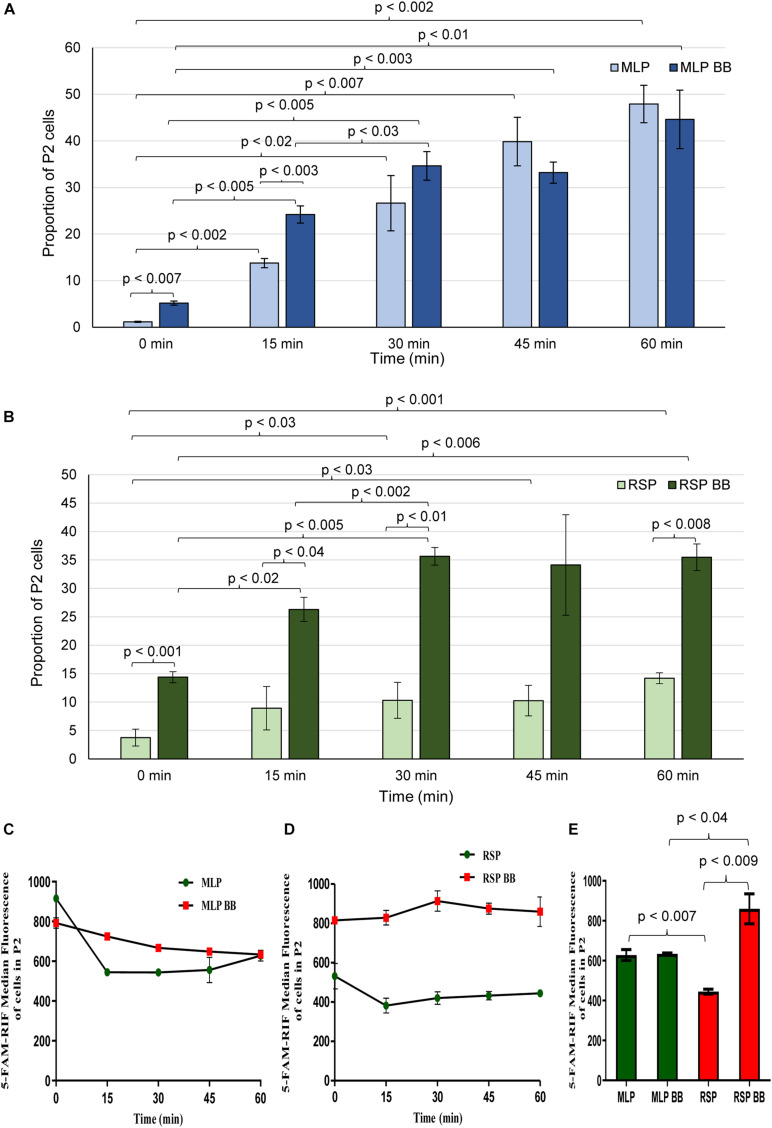
Flow cytometric analysis of 5-FAM-rifampicin permeability into *Mtb* MLP and RSP cells before and after bead beating. Bar graph showing the proportion of MLP and RSP cells in the P2 gate during 60 min of time-dependent permeability of 5-FAM-rifampicin into: **(A)** MLP cells and **(B)** RSP cells, before and after bead beating. **(C,D)** Time-dependent presence of 5-FAM-rifampicin fluorescence in the P2 gated population of: **(C)** MLP and **(D)** RSP cells exposed to 5-FAM-rifampicin for 60 min. **(E)** Quantitative significant difference between the 5-FAM-rifampicin median fluorescence of the P2 gated population of MLP and RSP cells at 60 min, before and after bead beating. Statistical significance was calculated using paired *t-*test (*n* = 3).

The extent of 5-FAM-rifampicin permeability into the cells of the rifampicin surviving population was compared with that of the MLP cells using flow cytometry. For this purpose, the MLP cells and the cells of the rifampicin surviving population were incubated with 1.5 μg/ml of 5-FAM-rifampicin for 1 h before and after bead-beating. At every 15 min, aliquots were collected and analyzed using flow cytometry to calculate the extent of the intensity of 5-FAM-rifampicin fluorescence in the cells. The MLP cells, before and after bead-beating, showed comparable profile with a steady increase in terms of the 5-FAM-rifampicin fluorescence in a time-dependent manner (Figures 2A,C,E and Supplementary Figures 3, 4, 5A). It showed that the permeability of 5-FAM-rifampicin was not affected by the capsular outer layer of the MLP cells. On the contrary, after bead beating, the cells of the rifampicin surviving population showed significant increase in the intracellular fluorescence of 5-FAM-rifampicin during the first 30 min of incubation, which was followed by a plateau (Figures 2B,D,E and Supplementary Figures 5B, 6, 7). It suggested that the presence of an altered capsular outer layer, which might have functioned as a ‘physical barrier’ to restrict 5-FAM-rifampicin permeability, might have been the reason for the reduced 5-FAM-rifampicin fluorescence in the cells of the rifampicin surviving population.

### The Hydrophilic Surface of the Cells of the Rifampicin Surviving Population

**FIGURE 3:**
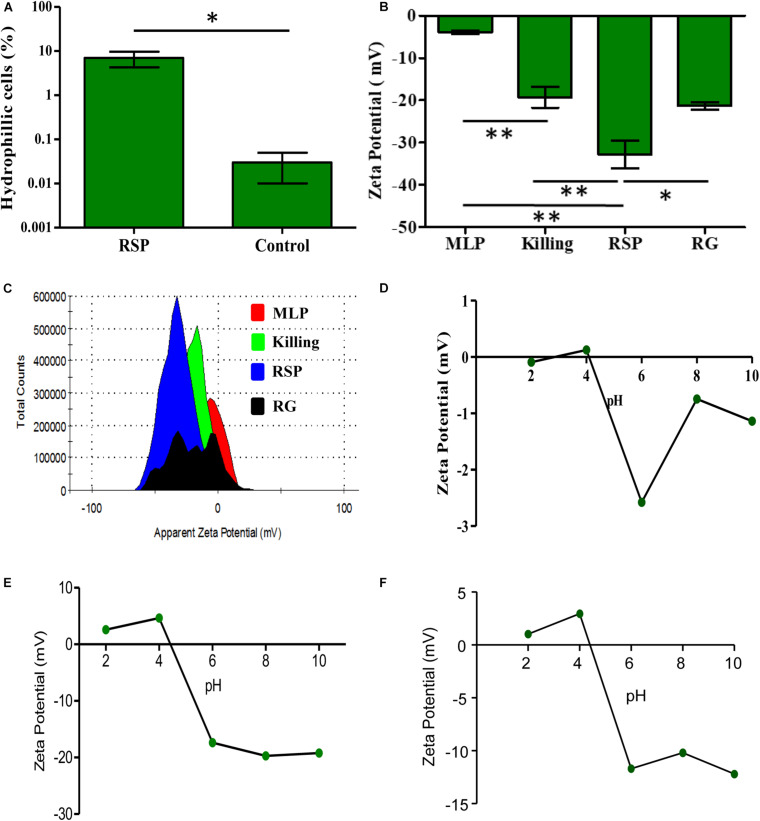
Physicochemical properties of rifampicin-exposed *M. tuberculosis* cells from the rifampicin surviving population. **(A)** Hexadecane assay showing the proportions of hydrophilic cells of the rifampicin surviving population and MLP cells. **(B)** ZP of cells from different phases of rifampicin exposed of *M. tuberculosis* cells and its corresponding **(C)** histogram overlay. ZP of the cells as a function of pH showing the isoelectric point of the cells between pH 4.0 and pH 5.0 for: **(D)** MLP cells, **(E)** the cells of the rifampicin surviving population, and **(F)** cells from killing phase. One asterisk (^∗^) indicates *P*-value less than or equal to 0.05 (*P* ≤ 0.05). Two asterisks (^∗∗^) indicate *P*-value less than or equal to 0.01 (*P* ≤ 0.01). The statistical significance was calculated using two-tailed paired *t*-test.

The considerable decrease in the rifampicin permeability imposed by the capsular outer layer on the *Mtb* cells of the rifampicin surviving population could be due to several reasons that may be reflective of the biophysical properties and/or composition of the capsular outer layer. Therefore, to understand how capsular outer layer reduces rifampicin permeability, the surface properties of the cells from the rifampicin surviving population and the molecular composition of the capsular outer layer were studied. As part of this attempt, their cell-surface hydrophobicity was measured in comparison to that of the MLP cells using hexadecane hydrophobicity assay for bacterial cells, as previously reported (Rosenberg et al., 1980). In this assay, the hydrophobic-surfaced and hydrophilic-surfaced cells of the rifampicin surviving population and the MLP population were partitioned between the hydrophobic hexadecane and aqueous buffer. After phase partitioning, the proportion of the cells retained in the aqueous layer was determined by plating aliquots of the aqueous phase. The CFU obtained was compared to the CFU of the total input cells to estimate the fraction of the cells with hydrophilic surface. About 10% of the cells of the rifampicin surviving population was retained in the aqueous phase, as compared to about 0.03% of MLP cells (Figure 3A). Thus, the proportion of the cells having hydrophilic surface was about 233-fold higher in the rifampicin surviving population as compared to those in the MLP population. The higher proportion of the cells with hydrophilic nature of the surface of the cells of the rifampicin surviving population suggested that exposure to lethal doses of rifampicin generated a population of cells having higher hydrophilic surface charge, probably reflected by different composition of the capsular outer layer, as compared to those of the cells in the MLP population.

### Negatively Charged Surface of the *Mtb* Cells of the Rifampicin Surviving Population

Having found that the surface of the cells in the rifampicin surviving population is more hydrophilic than that of the MLP cells, we determined their net surface charge in comparison to that of the MLP cells. It was earlier reported that the capsular outer layer of actively growing mycobacteria mostly contained neutral polysaccharides and proteins, besides low amounts of lipids (Ortalo-Magne et al., 1995). We hypothesized that the hydrophilic nature of the surface of the cells of the rifampicin surviving population might be due to the presence of charged polysaccharides in the capsular outer layer. Therefore, we determined the net charge of the cell surface by measuring the zeta potential (ZP) of the cells in terms of the electrophoretic mobility of the cells in liquid medium, as described (Wilson et al., 2001; Ayala-Torres et al., 2014). The respective ZP values of the *Mtb* cells from the MLP, the killing phase, rifampicin surviving population and the regrowth phase were determined individually and compared.

Upon prolonged exposure to rifampicin, the ZP value of (−)3.91 mV of the *Mtb* MLP cells gradually increased to (−)19.26 mV on the cells in the killing phase, and to (−)32.76 mV on the cells from the rifampicin surviving population (Figure 3B). Such an increase implied that the cells in the rifampicin surviving population have significantly higher negative charge on their surface as compared to that of the cells from the other phases of rifampicin exposure. The significantly high negative surface charge of the cells in the rifampicin surviving population implied the accumulation of negatively charged (anionic) molecules on the cells’ surface. The significant ZP value of (−)21 mV of the regrowth (RG) phase cells probably suggested that the content of the negatively charged molecules on the surface of the regrowing cells might have begun to decline due to resumption of growth and division of a subpopulation of the cells, enabled by gain of genotypic resistance to rifampicin. In fact, we had earlier demonstrated that rifampicin resistant genetic mutants emerged *de novo* from the rifampicin/moxifloxacin surviving populations of *Mtb* and *Msm* exposed to the antibiotics for prolonged durations (Sebastian et al., 2017; Swaminath et al., 2020a, b). Consistent with these possibilities, the ZP histogram of the RG phase population showed multiple peaks, suggesting the existence of heterogeneity in terms of the extents of the contents and the negative charge of the capsular outer layer the regrowing population due to resumed growth and cell division (Figure 3C).

### Ionic Properties of the TOL of the Cells From the Rifampicin Surviving Population

Since we observed high negative charge on the surface of the cells from the rifampicin surviving population, we further sought to determine the ionic properties of the cell surface. For this, we compared the isoelectric point (pI), which is the pH at which the net surface charge of the cells (ZP) becomes zero, of the *Mtb* cells of the rifampicin surviving population, in comparison to the pI values of the cells in the killing phase and MLP over the pH values 2, 4, 6, 8, and 10. The near-to-zero ZP values of MLP cells at pH 2.0 and pH 4.0 declined to (−)2.58 mV at pH 6.0, suggested that the MLP cells have a pI between pH 4.0 and pH 6.0 (Figure 3D). The cells of the rifampicin surviving population also showed a similar pI profile, with their pI closer to pH 4.0, and with lower ZP values of (−)20 mV at higher pH (Figure 3E). The killing phase cells also had a pI between pH 4.0 and pH 6.0, with the value being closer to pH 4.0. However, the decline of the negative ZP value at higher pH was considerably lesser for the killing phase cells than that for the cells of the rifampicin surviving population (Figure 3F, compare with Figure 3E). Thus, the *Mtb* cells from the MLP, killing phase and rifampicin surviving population showed comparable pI values, which probably indicated that the nature of the negatively charged molecules might not have changed on the cells’ surface during the transition from the killing phase to the rifampicin surviving population and to the regrowth phase. But rather, the content of those molecules might have increased on the cells of the rifampicin surviving population, compared to the cells of other phases. However, the higher negative ZP value of the cells of the rifampicin surviving population at higher pH values, unlike of the killing phase or MLP cells, indicated higher negative charge on the surface of these cells. This could be possibly due to the higher content of negatively charged molecules on the surface of the cells of the rifampicin surviving population as compared to those on the surface of the cells from the MLP or killing/regrowth phase. These observations prompted us to examine the ultrastructure of the cells of the rifampicin surviving population using transmission electron microscopy.

### Thickened Capsular Outer Layer on the Cells in the Rifampicin Surviving Population

**FIGURE 4:**
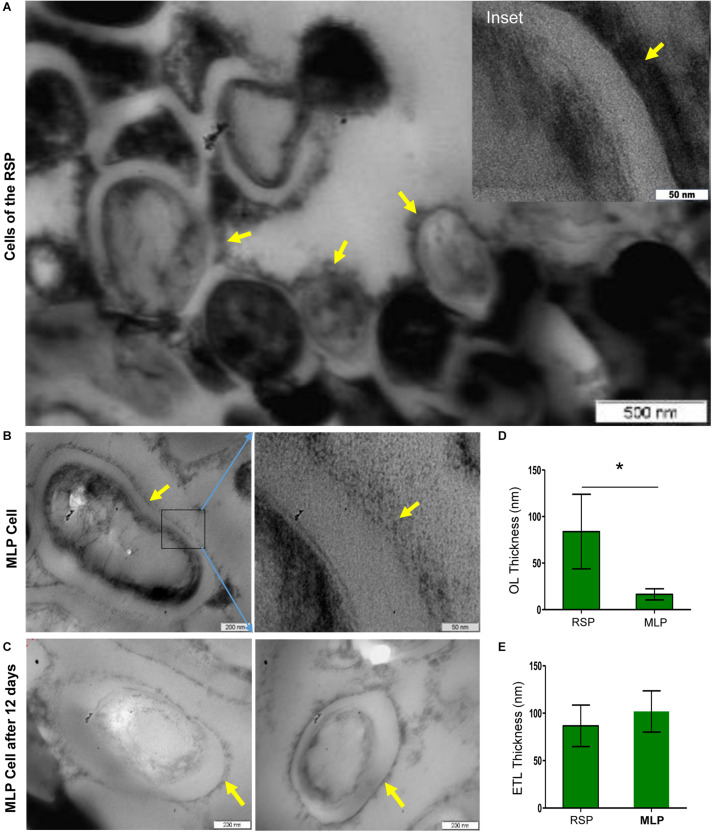
Presence of TCOL in the *M. tuberculosis* cells from the rifampicin surviving population (RSP). **(A,B)** Transmission electron micrograph of the cells of the rifampicin surviving population (RSP; 12th day of exposure) and of the MLP population. **(A)** The cells of the RSP with thickened capsular outer layer (TCOL). **(B)** The MLP cell (control) with NCOL. Magnified images are shown as inset in **(A)** and on the respective adjacent panels in **(B)**. The NCOL of the MLP cells cultured for 12 days without rifampicin is shown in **(C)**. The yellow arrows indicate TCOL/NCOL of RSP/MLP, respectively. **(D)** Thickness (in nm) of the TCOL/NCOL of the cells of the RSP and MLP population. ^∗^ indicates *p* value <0.05. **(E)** Thickness (in nm) of electron transparent layer (ETL) of the cells of the RSP and MLP populations.

Due to the reasons mentioned under the section, ‘Experimental Rationale and Strategy’, the whole of the rifampicin surviving population was taken for determining ultrastructure of the cells. Transmission electron micrographs (TEM) of the cells of the rifampicin surviving population showed that the capsular outer layer was thick, uneven, loosely bound and deeply stained, which we called thickened capsular outer layer, TCOL (Figure 4A with the inset image of the TCOL). Since the whole of the rifampicin surviving population was taken for the analysis, as expected, we could observe tannic acid positive darkened dead/damaged cells (Figure 4A). On the contrary, the MLP cells (control sample) possessed a light evenly stained normal capsular outer layer, called NCOL (Figure 4B), as reported earlier (Ortalo-Magne et al., 1995). The MLP cells, which were cultured for 12 days in the absence of rifampicin, which was the rifampicin-unexposed control sample equivalent to the duration of the MLP cells exposed to rifampicin for 12 days, did not develop TCOL (Figure 4C). More images in Supplementary Figure 8. While the average thickness of NCOL was 15 ± 10 nm, the thickness of the TCOL was found to be 90 ± 40 nm, which was significantly higher than that of the NCOL (Figure 4D). The electron transparent layer (ETL) of the cells of the rifampicin surviving population and the MLP possessed comparable thickness (Figure 4E), as previously reported (Rastogi et al., 1986). It indicated that the ultrastructural difference between the cells of the rifampicin surviving population and the MLP was confined to the thickness of the capsular outer layer. Further, the tannic acid negative cells, which are live cells (Núnez-Durán, 1980; Stirling, 1993), alone were considered for the analysis in the study. Since some of the tannic acid positive cells also showed TCOL, we did not estimate the proportion of the cells with TCOL. Nevertheless, a small proportion of the cells in the rifampicin surviving population did possess TCOL.

### Nature of the Constituents of the TCOL

It was earlier reported that the outer capsular material of the actively growing *Mtb* was constituted majorly by polysaccharides and proteins, besides small amounts of lipids (Ortalo-Magne et al., 1995). The capsular outer layer is rich in polysaccharides constituted by D-glucan, D-arabino-D-mannan, and D-mannan which contain glucose mannose residues (Rastogi et al., 1986; Lemassu and Daffé, 1994; Ortalo-Magne et al., 1995; Stokes et al., 2004; Kalscheuer et al., 2019). A high molecular weight α-glucan (>100 kDa), which constitutes up to 80% of the capsular outer layer of *Mtb* cells, was found to contain glucose units in →4-α-D-Glc-1→ core with branching at position 6 at every 5 or 6 residues with →4-α-D-Glc-1→ oligoglucosides (Lemassu and Daffé, 1994; Ortalo-Magne et al., 1995; Dinadayala et al., 2004). The terminal residues were found to be Glc*p*-(1 → 4)-, Glc*p*-(1 → 6)-, and (→4)-Glc*p*-(1 → 6)- (Dinadayala et al., 2004). The fluorophore, calcofluor white (CFW), specifically binds the β-configuration glucose units, glucose-β-(1 → 4)-mannose, glucose-β-(1 → 4)-glucose, and glucose-β-(1 → 3)-glucose, in polysaccharides (Maeda and Ishida, 1967; Wood, 1980), rather than the α-configuration units.

**FIGURE 5:**
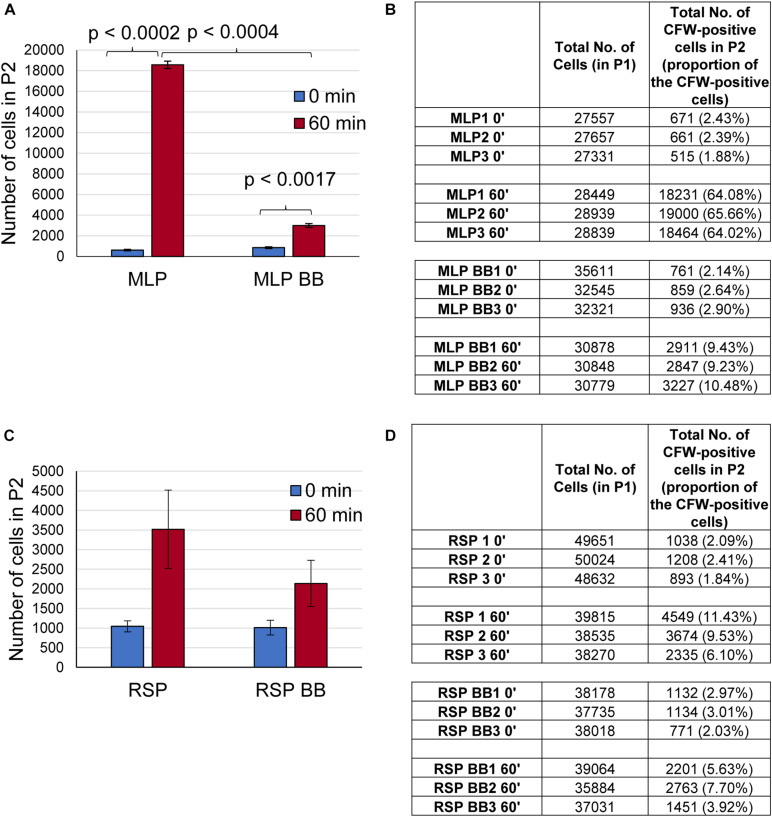
Presence of polysaccharide on the surface of the cells of the rifampicin surviving population (RSP). **(A–D)** Quantitation of the flow cytometric analysis of calcofluor white (CFW) stained *Mtb* MLP and RSP cells before and after bead beating. Bar graph and table showing the number of cells of the: **(A,B)** MLP and **(C,D)** RSP in the P2 gate after 60 min of CFW staining. The proportions of the respective cells are indicated in parenthesis. Statistical significance was calculated using paired *t-*test (*n* = 3).

Flow cytometry analysis showed that ∼64–65% of the CFW-stained MLP cells (control sample) at 0 min got stained in 60 min of incubation (Figures 5A,B; see P2 gate in Supplementary Figures 9A,B). However, only ∼9–10% of the MLP cells at 60 min retained CFW fluorescence after bead beating (Figures 5A,B; see P2 gate in Supplementary Figures 9C,D). On the contrary, only ∼6–11% of the cells of the rifampicin surviving population (RSP) at 0 min got stained during the 60 min of incubation (Figures 5C,D; see P2 gate in Supplementary Figures 10A,B). After bead beating, ∼3.9–7.7% of the RSP cells retained CFW fluorescence even (Figures 5C,D; see P2 gate in Supplementary Figures 10C,D). These observations indicated the presence of significantly higher levels of polysaccharide containing CFW-specific glucose/mannose units in β (1 → 4) or β (1 → 3) linkages on the surface of the MLP cells than in the capsular outer layer of the cells in the rifampicin surviving population. However, a larger proportion of the RSP cells remaining stained with CFW, as compared to that of MLP cells, probably might be due to the significantly higher thickness of the TCOL as compared to the thin NCOL. Since all the earlier studies described above have shown the capsular layer of *Mtb* cells containing glucose-α-(1 → 4)-glucose linkages, which is not bound by CFW (Maeda and Ishida, 1967; Wood, 1980), the proportion of polysaccharides containing glucose/mannose units in β (1 → 4) or β (1 → 3) linkages might be significantly higher in the NCOL of MLP cells than in the TCOL of the cells of the rifampicin surviving population.

### Molecular Composition of the TCOL

The TCOL and NCOL, gently extracted, as reported previously (Ortalo-Magne et al., 1995), were used for GC-MS analysis to identify the type of the polysaccharides in them. The fold-change in the relative abundance of the monosaccharides detected in the TCOL, with respect to those in the NCOL, were listed (Table 1; raw data in Supplementary Table 1). The composition of NCOL was comparable with an earlier study on the NCOL of actively growing *Mtb* cells (Ortalo-Magne et al., 1995). However, the levels of these constituents in the TCOL of the cells from the rifampicin surviving population were found to be several fold higher than in the NCOL (Table 1). Besides α-D-glucopyranosides, levels showed six–sevenfold increase. The higher levels of α-D-glucopyranoside indicated the possibility of the presence of trehalose, which is composed of two units of α-D-glucopyranoside, as α-D-glucopyranosyl-α-D-glucopyranoside. A high content of glucan also could be an indication of the high levels of α-D-glucopyranoside. The presence of about twofold higher levels of arabinose and mannose could also indicate the presence of arabinomannan, which has been found to be a significant component of NCOL in the mycobacterial MLP cells (Lemassu and Daffé, 1994; Daffé and Etienne, 1999; Jankute et al., 2015). The higher levels of β configuration sugars in the NCOL of MLP cells (see Supplementary Table 1) correlated with the higher CFW binding onto the MLP cells. Thus, while the composition of the NCOL and TCOL were comparable between the MLP and the cells from the rifampicin surviving population, the TCOL contained significantly higher levels of the components.

**TABLE 1:** Relative abundance of the components of TCOL^a^ in the cells from the rifampicin surviving population with respect to those of NCOL^b^ in the MLP^c^ cells, as analysed using GC-MS.

Component	^d^Relative fold-increase in the RSP cells (w.r.t. MLP cells)
α-D-glucopyranoside	6.67
1, 2, 5-linked-mannitol	4.97
3, 4-linked mannose	2.22
Hexa-acetyl-mannitol	1.84
1, 2, 3-propanetriol	1.83
Methyl 1, 2, 3, 4-tetrahydronaphthalene-2-carboxylate	1.76
1, 3-di-iso-propylnaphthalene	1.73
1, 2, 4-linked arabinitol	1.68
D- (1, 2-linked mannitol)	1.58
1, 3-di-iso-propylnaphthalene	1.41
5-linked galactonitrile	1.30
Galactose pyranoside	1.11
1, 7-di-iso-propylnaphthalene	1.10
α- D-mannopyranoside	1.08

*TCOL^a^, thickened capsular outer layer; NCOL^b^, normal capsular outer layer; MLP^c^, mid-log phase. ^d^The percentage “relative abundance” of each compound in the capsular outer layer of MLP and rifampicin surviving population was independently determined from the GC-MS data. By comparing these values for each compound, the fold difference was calculated. Since it is a relative quantitation, normalization was not performed for the values with equal cell number/biomass or any internal standard.*

## Discussion

### Thickening of Capsular Outer Layer Restricting Rifampicin Entry in *Mtb* Cells

The present study showed that the *Mtb* cells in the rifampicin surviving population possessed a thickened capsular outer layer (TCOL) with an increased content of negatively charged polysaccharides that conferred high negative charge on the cell surface. The increased thickness and the high negative charge of the TCOL might have rendered it a ‘physical barrier’ that reduced permeability of the relatively non-polar 5-FAM-rifampicin into the *Mtb* cells. The lack of TCOL in the *Mtb* H_37_R_a_ MLP cells even after 12 days of culture ensured that the development of TCOL in the cells of the rifampicin surviving population was a specific mechanism adopted only when the bacilli encountered rifampicin to reduce intracellular rifampicin concentration. We recently found that the non-replicating persistent (NRP) stage 2 (hypoxic) *Mtb* H_37_R_a_ cells in Wayne’s *in vitro* hypoxia model of dormancy also possessed thickened capsular outer layer, which was lost upon shifting of the NRP stage 2 cells from hypoxia to normoxia was (Jakkala and Ajitkumar, 2019).

These observations suggested that *Mtb* cells showed similar response to antibiotic and hypoxia stress conditions to reduce intracellular rifampicin concentration for their survival under the stress conditions. Further, the ultrastructure of the *Mtb* cells from both the NRP stage and the rifampicin surviving population revealed that the cell-wall change was confined to the capsular outer layer only and not to the peptidoglycan layer or the electron transparent layer. Alterations in the cell-wall, which resulted in the abolition of acid fastness and rod shape, have been found in the nutritionally starved mycobacterial cells (Nyka, 1974). Although nutritional stress alone did not cause thickening of capsular outer layer. As revealed by the control sample in the present study, nutritional stress combined with low oxygen or gradual acidification had often resulted in the thickening of cell-wall (Cunningham and Spreadbury, 1998; Shleeva et al., 2011). It has been recently shown that even phenotypic antibiotic tolerance, such as the saline-induced antibacterial tolerance, involved cell envelope remodeling independent of the metabolic changes and growth arrest associated with the stress (Larrouy-Maumus et al., 2016).

### Tween 80 and Dead Cells Do Not Affect Rifampicin Permeability Assays

Tween 80, even as low as 0.05%, has been found to improve the permeability of many antituberculosis drugs, including rifampicin, in *Mycobacterium intracellulare*, *M. smegmatis*, and *Mycobacterium avium complex* and thereby reduce the CFU against the drugs (Hui et al., 1977; Masaki et al., 1990; Yamori and Tsukamura, 1991). The hydrolysis of Tween 80 liberated oleic acid that got accumulated both at the cell surface and in the macromolecular lipid fraction (Schaefer and Lewis, 1965; Stinson and Solotorovsky, 1971). Therefore, Tween 80 in the growth medium could indirectly influence the cells’ permeability to rifampicin. In our study, between the 12-day old rifampicin-exposed cells of the rifampicin surviving population and the 12-day old rifampicin-unexposed cells, only the former developed TCOL but not the latter, despite both containing 0.05% Tween 80. Further, even with Tween 80 in the medium, the cells of the rifampicin surviving population showed reduced rifampicin permeability. These differences between the two types of cultures ruled out the possibility of Tween 80 having any specific influence on the capsular outer layer modification exclusively on the cells from the rifampicin surviving population but not on the 12-day old rifampicin-unexposed cells.

The rifampicin surviving population was found to contain large number of dead cells, as expected due to the initial killing phase. For the reasons described under ‘Experimental Rationale and Strategy,’ it was not possible to isolate the live cells alone from the rifampicin surviving population. Although single cell studies were possibilities, we did not attempt them as they would not have given the global picture of the processes in the whole population of rifampicin surviving cells. Further, we did not also use microfluidics to study the phenomenon as it would have added the mechanical pressure and the isolated status of the cells as additional stress conditions. Such stress conditions might inflict their own effects on the cells, which would have complicated the interpretations. Further, under many stress conditions, bacterial subpopulations elicit co-operation, as we found between two subpopulations in the whole culture exposed to rifampicin (Nair et al., 2019). Such natural co-operative processes between subpopulations in the rifampicin surviving population would not be operational in microfluidics experiments.

### The Features of TCOL That Might Have Reduced Rifampicin Permeability

Several lines of evidence from the present study suggest that the features of TCOL that might have restricted permeability to rifampicin were most probably the significantly high thickness and the elevated negative charge on the surface of the cells of the rifampicin surviving population due to the accumulation of high levels of negatively charged polysaccharides.

#### Contribution of the Thickness

The high levels (six–sevenfold) of α-D-glucopyranoside and fivefold increase in the 1, 2, 5-linked-Mannitol in the TCOL implied that their content in the MLP cells might have increased when the cells reached the rifampicin surviving phase. The increased levels of α-D-glucopyranoside, the units each of which constitute the disaccharide, trehalose (α-D-glucopyranosyl-α-D-glucopyranoside), indicated that the TCOL might contain these molecules which protect bacterial cells against severe stress conditions and desiccation (Argüelles, 2000; Elbein et al., 2003). In fact, reduced levels of trehalose dimycolates (TDMs) had been found to contribute to enhanced sensitivity to multiple antibiotics (Nguyen et al., 2005). Similarly, since glucan is a polymer of α-D-glucopyranoside, the abundant presence of α-D-glucopyranoside indicated the presence of glucan also. It has been found to be a constituent of the *Mtb* cell surface molecules and it is expressed in *Mtb* cells cultured *in vitro* and *in vivo* (Schwebach et al., 2002). Further, the polysaccharide layer has been found to have about two-orders of magnitude lesser permeability to lipophilic probes as compared to the permeability of the conventional phospholipid bilayer (Plésiat and Nikaido, 1992). Reduced antibiotic permeability has also been observed due to the presence of altered LPS chains with O-chains (Robbins et al., 2001). In view of all these studies, the significant increase in the content of polysaccharides might have contributed to the thickening of the capsular outer layer, even though the thickness was non-uniform and the TCOL was loosely bound to the cell surface. The thickened capsular outer layer (TCOL) of the cells in the rifampicin surviving population might have put up a ‘physical barrier’ to rifampicin, thereby restricting the entry of the antibiotic.

#### The Role of Cell Surface Negative Charge

The accumulation of negatively charged capsular OL components (essentially polysaccharides) might have caused significant increase in the negative charge density on the surface of the cells from the rifampicin surviving population. The higher negative charge (conferring polar nature) might have reduced the permeability of the relatively non-polar 5-FAM-rifampicin. However, it was reported that rifampicin, rifapentine, bedaquiline, clofazimine, and nitrazoxamide, which are relatively non-polar, lipophilic antibiotics, reduced the CFU of the *Mtb* cells under hypoxia by ≥2-log_10_ in Wayne’s *in vitro* hypoxia model with modifications wherein the cells were cultured at pH 5.8 (Piccaro et al., 2015). On the contrary, pyrazinamide, moxifloxacin, isoniazid, ethambutol, metronidazole, meropenem, which are relatively more polar and hydrophilic in nature, did not cause reduction in the CFU (Piccaro et al., 2015). This observation might imply that the restricted entry of rifampicin, which is relatively non-polar, cannot be attributed to the polar and hydrophilic property of the TCOL. However, it was possible that the low pH (5.8) might have protonated the negative charges on the surface, thereby conferring a relatively non-polar nature to the cells’ surface. This in turn might have facilitated the permeability of non-polar rifampicin and other lipophilic drugs, but not of the hydrophilic drugs. On the contrary, in our study, the elevated negative zeta potential values revealed that at the pH 7 of Middlebrook 7H9 medium in which the cells of the rifampicin surviving population were present, the cells’ surface might still be remaining negatively charged. This in turn would have restricted the permeability of the relatively non-polar 5-FAM-rifampicin. Thus, a combined effect of the increased thickness and the highly anionic nature of the TCOL might provide a ‘negatively charged physical barrier’ restricting the permeability of rifampicin into the *Mtb* cells of the rifampicin surviving population. The significant thickening of the capsular OL with elevated negative surface charge might be a common adaptive feature of *Mtb* cells to survive against antibiotics (present study) and as dormant cells under hypoxia (Jakkala and Ajitkumar, 2019).

#### Some Unique Constituents of TCOL

The presence of α-D-glucopyranoside was an indication of the presence of trehalose and glucan, which have been found to be part of the *Mtb* envelope (Sambou et al., 2008; Chiaradia et al., 2017; Kalscheuer et al., 2019). Further, an abundance of α-D-glucopyranoside suggested elevated levels of trehalose and glucan, either of which has been implicated as protective agents for the survival of bacteria under severe stress conditions (Argüelles, 2000; Elbein et al., 2003) and during persistent infection of *Mtb* in mice (Sambou et al., 2008). Thus, the presence of probably high levels of trehalose and glucan, which can protect the cells from severe stress conditions, is consistent with the severe stress condition of survival under the lethal concentrations of rifampicin.

### Formation of TCOL in the Avirulent *Mtb* H37R_a_ Cells

It is known that the transcriptional regulator, PhoP, in *Mtb* H_37_R_v_ is required for the regulation of the secretion of ESAT-6, specific T-cell recognition, and the synthesis of polyketide-derived lipids, and thereby play major roles in several cellular processes (Pérez et al., 2001; Gonzalo-Asensio et al., 2006; Chesne-Seck et al., 2008; Frigui et al., 2008). The PhoP mutation in *Mtb* H_37_R_a_, which we used as the experimental system to study the capsular OL thickening in cells from the rifampicin surviving population, contributes to the avirulence of the strain (Lee et al., 2008). Hence it could be argued that *Mtb* H_37_R_v_ cells from the rifampicin surviving population might not develop TCOL, and if at all they could show thickening of capsular OL, the composition of the TCOL might be different from that in *Mtb* H_37_R_a_, owing to the presence of a functional PhoP. However, there are studies that suggest that the capsular OL thickening, upon exposure to antibiotics, is not exclusively dependent on PhoP regulated gene products. For instance, *Mtb* multi-drug (MDR) and/or extensively drug-resistant (XDR) clinical isolates showed significant difference in the cell-wall thickness in comparison to that of the antibiotic-susceptible strains of mycobacterial isolates (Velayati et al., 2009a). While the cell-wall thickening in MDR strains was due to thickening of capsular OL and ETL, it was due to the thickening and fusion of the basal peptidoglycan layer with the ETL and in the XDR strains. About 5–7% of the XDR-TB isolates showed extraordinarily thick cell-wall (Velayati et al., 2009a). In the isolates of totally drug-resistant (TDR) strains, irrespective of genotype patterns or superfamilies of the strains, bacilli with extraordinarily thick cell-wall could be observed (Velayati et al., 2009b). Thus, it is interesting to note that the MDR, XDR and TDR bacilli, despite having functional PhoP, showed remarkable thickness of cell-wall, including that of the capsular OL, like the rifampicin-resistant cells from the rifampicin surviving population of the PhoP mutant laboratory strain, *Mtb* H_37_R_a_.

Secondly, like the cell-wall thickening in the *Mtb* H_37_R_v_ cells exposed to hypoxia for 18 months (Velayati et al., 2011), the hypoxia-exposed NRP stage 2 cells of *Mtb* H_37_R_a_ were found to possess TCOL, which we had called TOL in that work (Jakkala and Ajitkumar, 2019). The similar response of *Mtb* H_37_R_v_, possessing functional PhoP, and *Mtb* H_37_R_a_, having non-functional PhoP, showing TCOL under hypoxic condition and under drug resistance, showed that the thickening of capsular OL is not majorly dependent on PhoP regulons. It is also possible that alternate pathways may exist for cell-wall thickening despite a non-functional PhoP. Further, despite having functionally intact PhoP ortholog and its regulons, the saprophytic *Msm* cells did not develop TOL under hypoxia at which the PhoP mutant *Mtb* H_37_R_a_ showed TOL (Jakkala and Ajitkumar, 2019), despite the hypoxic responses of *Msm* and *Mtb* H_37_R_v_ being comparable (Dick et al., 1998). Above all, like *Mtb* H_37_R_a_, *M. smegmatis* also form the rifampicin surviving population against rifampicin (Grant et al., 2012; Sebastian et al., 2017; Swaminath et al., 2020b). These diverse studies support that the observations made on the development of TCOL in the cells of the rifampicin surviving population of *Mtb* H_37_R_a_ are meaningful and not unnatural, despite the non-functional PhoP, and therefore the conclusions drawn therefrom could be of physiological relevance.

### Demonstration of TCOL Using Conventional TEM

For the preparation of the samples for the ultrastructural analysis of the *Mtb* cells from the rifampicin surviving population, we used conventional TEM (Takade et al., 1983), instead of the freeze-substitution technique (Takade et al., 2003; Yamada et al., 2010), for the following reasons. Both these methods, although differ in sample preparation, gave identical results on the presence of TCOL on NRP stage 2 hypoxic *Mtb* H_37_R_a_ cells (Jakkala and Ajitkumar, 2019). Further, this method had been successfully used to study thickening of capsular OL in the mycobacterial cultures at stationary phase (Cunningham and Spreadbury, 1998), for distinguishing resistant strains from non-resistant strains of *Mtb* (Velayati et al., 2009a) and from 150-days old cultures that were generated from the stationary phase onset cultures (Shleeva et al., 2011), and to characterize *Mtb* strains that were deficient in exopolyphosphatase (Chuang et al., 2015). Earlier, we had used conventional TEM to study the ultrastructure of cell wall layers of *Mtb* and *Msm*, and it provided distinct images of the capsular outer layer (OL), peptidoglycan layer (PGL), electron transparent layer (ETL) and cell membrane (Vijay et al., 2012). However, the samples were prepared in a gentle way without disturbing the loosely bound TCOL, thereby avoiding the possibility of dislodging TCOL. In the present study, we found that the proportions of the cells with TCOL from the rifampicin surviving population and the MLP cells with NCOL were consistent and reproducible in biological triplicates. Therefore, the sample preparation for TEM could not have dislodged the seemingly loosely bound TCOL, as otherwise it would have caused variations in the proportions of the cells with TCOL.

### The Relevance of Fluorescent Rifampicin to Study Antibiotic Permeability in *Mtb*

The preparation of the samples for the permeability assay using 5-FAM-rifampicin involved washing of the cells once with ice-cold Middlebrook 7H9 broth to remove any 5-FAM-rifampicin non-specifically bound to TCOL. This procedure ensured that the quantitative determination of intracellular 5-FAM-rifampicin using flow cytometry was truly that of the levels of permeated 5-FAM-rifampicin. Since flow cytometry counts only intact cells as events, and not dead cells, even if one considers the possibility of the probe getting non-specifically bound to dead cells. Hence the fluorescence intensity of 5-FAM-rifampicin determined using flow cytometry would be a measure of its intracellular levels in the bacterial cells. In fact, the 5-FAM-rifampicin fluorescence values were comparable between the unwashed cells and washed cells of the NRP stage 2 (hypoxic) *Mtb* H_37_R_a_ cells that possessed TOL (Jakkala and Ajitkumar, 2019). These observations ensured that 5-FAM-rifampicin was not remaining non-specifically and externally bound to TOL, which otherwise would have given inconsistent and erroneous measurements in the biological triplicate samples. The consistent and reproducible values of 5-FAM fluorescence in the flow cytometry experiments ensured that the observed values corresponded to the intracellular 5-FAM-rifampicin. For these reasons, we did not attempt to perform separate quantitation of the intracellular levels of 5-FAM-rifampicin in cell lysates.

### The Benefit of TCOL to the Cells of the Rifampicin Surviving Population

**FIGURE 6:**
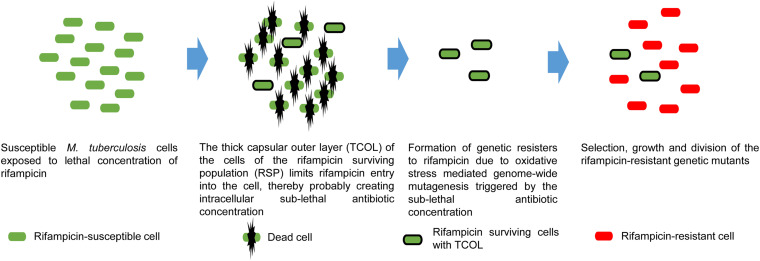
Proposed model on the benefit of TCOL in the generation of rifampicin genetic resisters from the *M. tuberculosis* cells of the rifampicin surviving population (RSP). *M. tuberculosis* cells (green) are exposed to lethal concentrations of rifampicin that kills susceptible cells. The cells of the RSP develop TCOL that restricts rifampicin permeability into the *M. tuberculosis* cells of the RSP, resulting in sub-lethal intracellular concentration of rifampicin. The presence of intracellular rifampicin invokes oxidative stress due to reactive oxygen species (hydroxyl radical) generation that inflicts genome-wide mutagenesis. This leads to the *de novo* emergence of antibiotic-resistant genetic mutants (red), as demonstrated by us (see Sebastian et al., 2017). The model represents a synthesis of the data from the present study and from Sebastian et al. (2017).

It was interesting to note that only ∼10% of the cells in the rifampicin surviving population possessed hydrophilic surface indicating the presence of negatively charged polysaccharides in the TCOL (see Figures 3A,B). It raised the question as to what would be the role of the TCOL if it is present only in ∼10% of the cells from the rifampicin surviving population? In this context, one may recollect the report that bacteria of diverse genera, upon exposure to sub-lethal concentrations of antibiotics, generate hydroxyl radical causing genome-wide mutagenesis that led to the emergence of antibiotic-resistant mutants regrowing in the presence of antibiotics (Kohanski et al., 2010; Long et al., 2016; Hoeksema et al., 2018). We recently reported that *Mtb* and *Msm* cells exposed to bactericidal concentrations of rifampicin and/or moxifloxacin also generated elevated levels of hydroxyl radical that resulted in the genome-wide mutagenesis causing the *de novo* emergence of rifampicin/moxifloxacin-resistant *rpoB/gyrA* mutants of *Mtb/Msm* from the rifampicin/moxifloxacin surviving population of cells in *in vitro* cultures (Sebastian et al., 2017; Swaminath et al., 2020a, b). Our present study has reconciled these two findings by demonstrating that the thickening of the capsular outer layer might have been a probable reason for the reduced intracellular concentration of rifampicin in the mycobacterial cells from the rifampicin surviving population. The reduced intracellular levels of the antibiotic in turn might have induced oxidative stress, enabling the emergence of rifampicin-resistant mutants. This could be the ultimate benefit of the TCOL in the cells of the rifampicin surviving population. Therefore, the presence of TCOL even in a low proportion of the cells from the rifampicin surviving population would facilitate the acquisition of antibiotic resistance thereby re-establishing a population of antibiotic-resisters. Based on the data from our present study, in the context of our earlier work (Sebastian et al., 2017; Swaminath et al., 2020a, b), these hypotheses were presented in a model (Figure 6).

### The Clinical Significance of the Study

In the treatment of tuberculosis, the antibiotics stay in the patients for a prolonged period as they get continuously replenished through the daily high dosage regimen. Hence there is a possibility that such prolonged exposure to high concentrations of rifampicin might generate the rifampicin surviving population with TCOL, as shown in the present study. This can bring about low intracellular rifampicin levels that can trigger the generation of ROS that can inflict genome-wide mutations (Sebastian et al., 2017; Swaminath et al., 2020a, b). In view of these possibilities, it is tempting to speculate that the presence of TCOL even in small proportions of the cells surviving in tuberculosis patients might contribute to the emergence of drug-resistant strains. Many earlier studies have shown the beneficial role of the capsular outer layer on various physiological events in mycobacteria (Armstrong and Hart, 1971; Saito et al., 1976; Lemassu and Daffé, 1994; Stokes et al., 2004; Ragas et al., 2007; Geurtsen et al., 2009). In this context, the present study on the TCOL imposing restricted permeability to rifampicin into the cells from the rifampicin surviving population of *Mtb* stands as an yet another example of the beneficial role of the capsular outer layer in bacterial physiology, enabling the emergence of rifampicin-resistant genetic mutants. Further, the phenomenon of the formation of TCOL on the *Mtb* cells of the rifampicin surviving population has clinical significance if it occurs in the tubercle bacilli in tuberculosis patients under treatment regimen with high doses of the relatively non-polar anti-tubercular antibiotic, rifampicin.

## Data Availability Statement

The original contributions generated for this study are included in the article/Supplementary Materials, further inquiries can be directed to the corresponding author.

## Author Contributions

PA and JS conceived and designed the experiments. JS, RRN, and SS performed the experiments. PA, JS, RRN, and SS analyzed the data. PA contributed reagents, materials, and analysis tools. PA and JS wrote the manuscript. PA, JS, RRN, and SS read and approved the manuscript. All authors contributed to the article and approved the submitted version.

## Conflict of Interest

The authors declare that the research was conducted in the absence of any commercial or financial relationships that could be construed as a potential conflict of interest.
