# Supplementary material for: Mycobacterium tuberculosis Cells Surviving in the Continued Presence of Bactericidal Concentrations of Rifampicin in vitro Develop Negatively Charged Thickened Capsular Outer Layer That Restricts Permeability to the Antibiotic
Source: Front Microbiol. 2020 Dec 17;11:554795. doi: 10.3389/fmicb.2020.554795 (PMC7773709; doi:10.3389/fmicb.2020.554795)
Supplement: Supplementary file 1 [file Data_Sheet_1.PDF]

SUPPLEMENTARY MATERIAL

FOR

Mycobacterium tuberculosis Cells Surviving in the Continued Presence of Bactericidal Concentrations of Rifampicin *In Vitro* Develop Negatively Charged Thickened Capsular Outer Layer that Restricts Permeability to the Antibiotic

Jees Sebastian, Rashmi Ravindran Nair[§], Sharmada Swaminath[§], Parthasarathi Ajitkumar*
Department of Microbiology and Cell Biology, Indian Institute of Science, Bangalore, Karnataka, India.

Present address: [†]Department of Medicine, Rutgers New Jersey Medical School, New Jersey, USA; [‡]Laboratory of Neurogenetics and Personalised Medicine, Nevada Institute of Personalised Medicine, UNLV School of Medicine, Las Vegas, NV, USA

[§]Contributed equally to this work

*Address correspondence to P. Ajitkumar, ajitkpartha@gmail.com

Running title: Restricted rifampicin entry in *Mycobacterium tuberculosis* rifampicin survivors

Supplementary Figures S1-S10

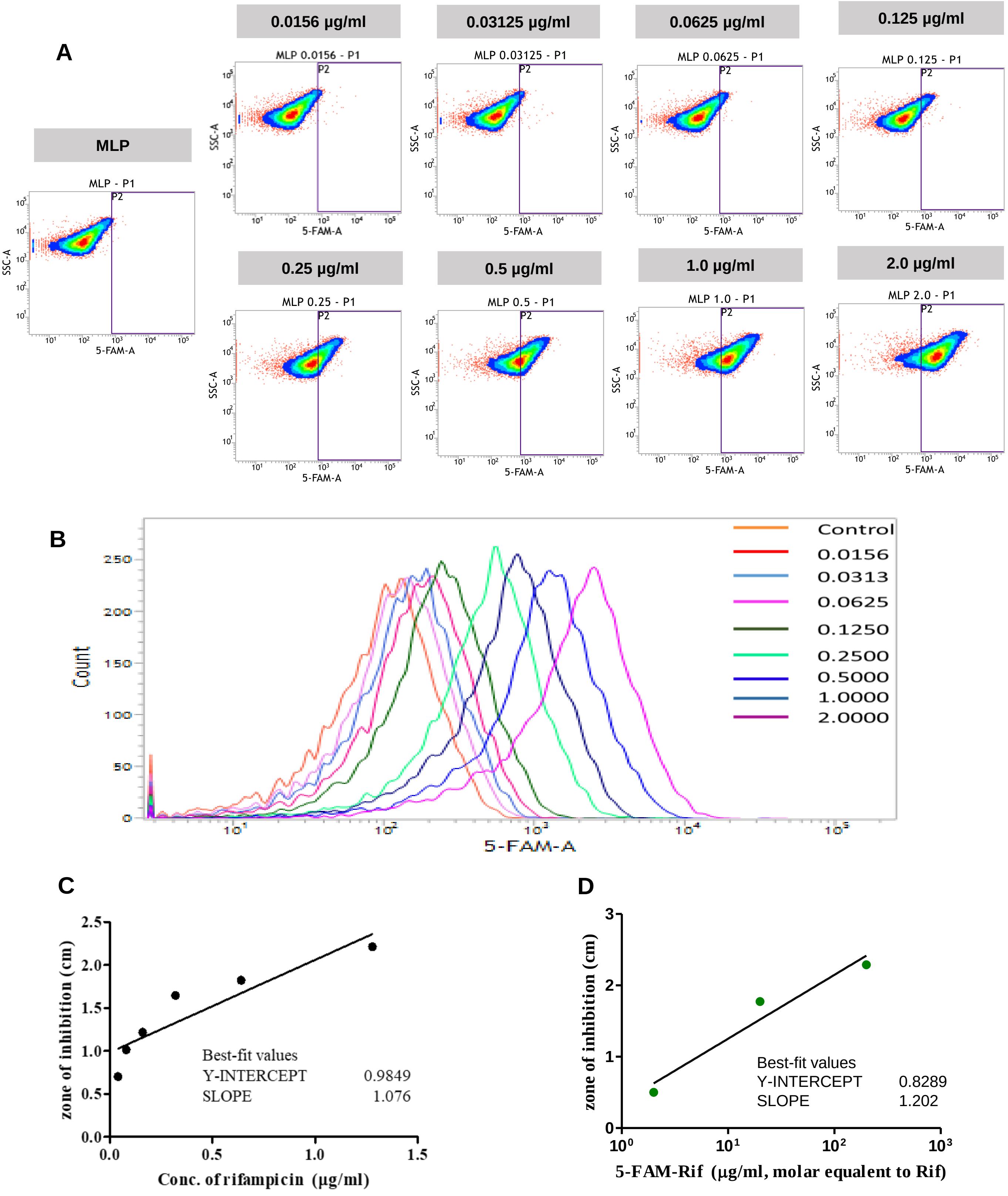

FIGURE S1. **Characterisation of 5-FAM-rifampicin permeability into MLP cells and bioassay of 5-FAM-rifampicin**. **(A, B)** Dose-dependent 5-FAM-rifampicin permeability into MLP cells. (**A**) Density plots and (**B**) histogram overlay of the flow cytometry profiles of MLP cells incubated with two-fold increasing concentrations of 5-FAM-rifampicin. **(C, D)** Bioassay for: **(C)** Rifampicin and (**D**) 5-FAM-rifampicin by agar diffusion method using rifampicin sensitive *Staphylococcus aureus* (ATCC 25923). The standard graph showing the diameter of zone of inhibition (Y-axis) against known concentrations of rifampicin (X-axis). Molar equivalent concentrations of 5-FAM-rifampicin (w.r.t. rifampicin) was used in the bioassay (see MATERIALS AND METHODS).

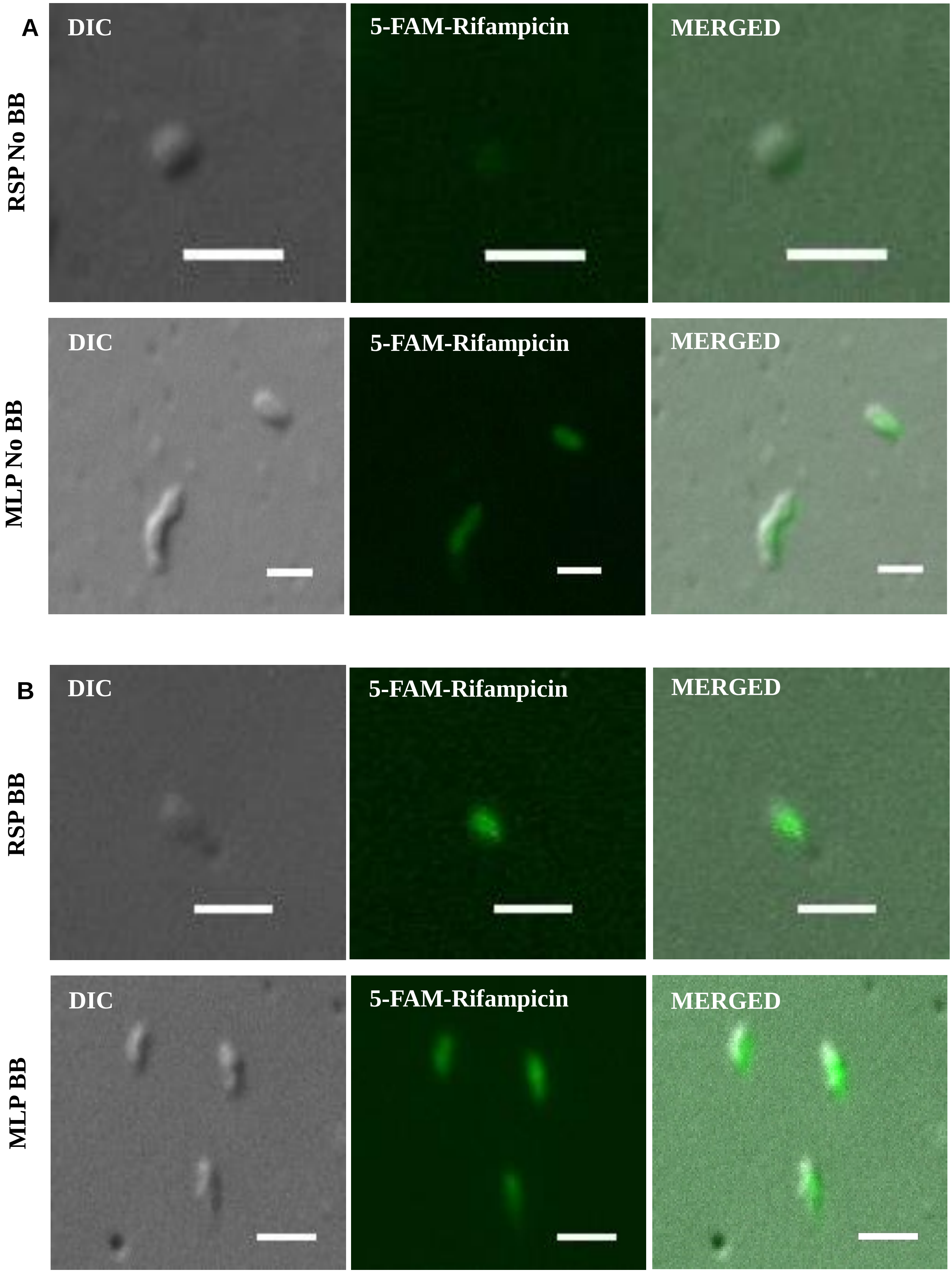

FIGURE S2. **Qualitative determination of 5-FAM-rifampicin permeability**. (**A, B**) Fluorescence microscopy images of *M. tuberculosis* cells of the rifampicin surviving population (RSP) and MLP showing differential entry of 5-FAM-rifampicn. RSP and MLP cells exposed to 5-FAM-rifampicin for one hr: (**A**) without bead beating; (**B**) after bead beating. Scale, 2 µm in each case as the RSP cells are smaller in size as compared to the MLP cells.

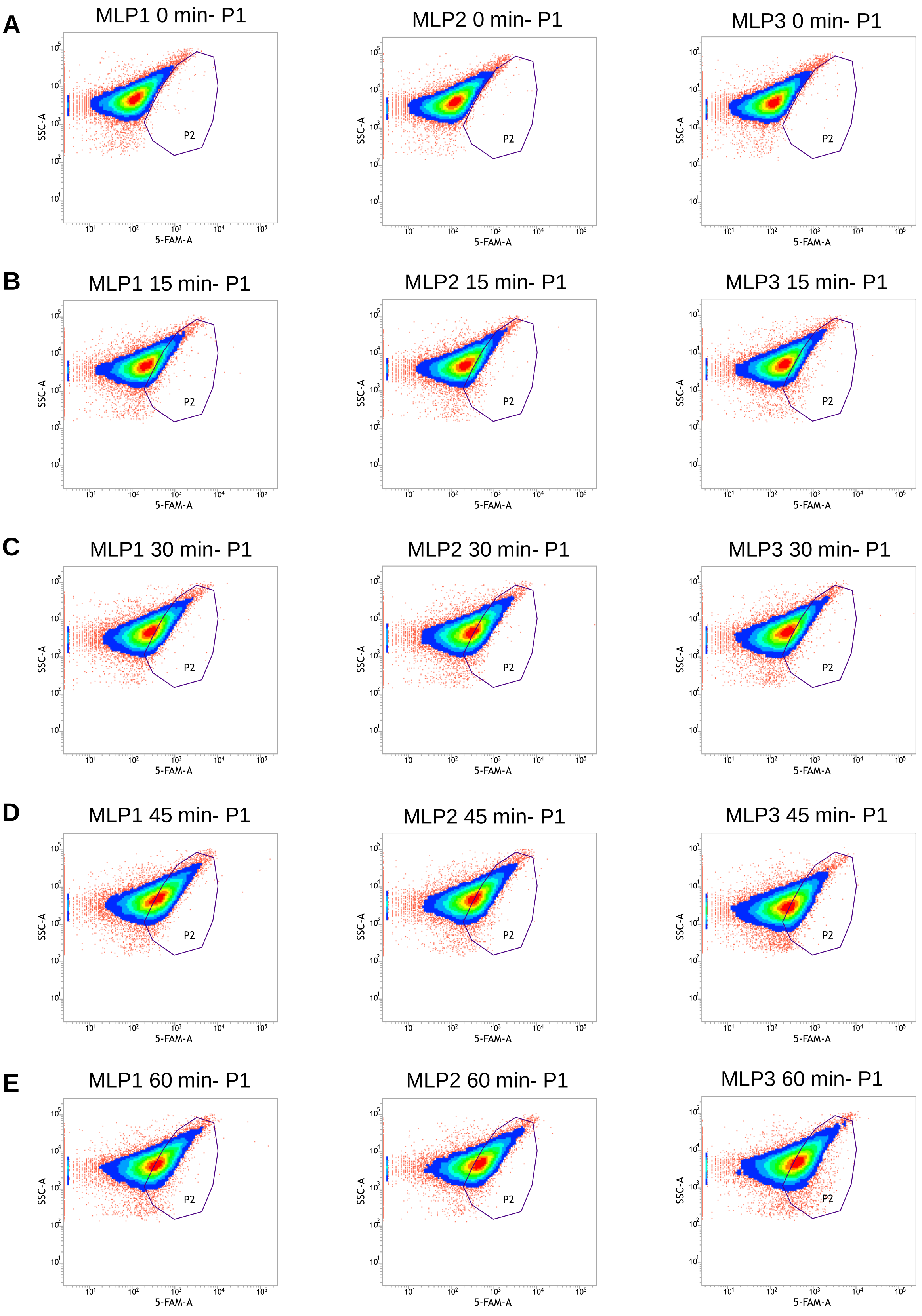

FIGURE S3. Flow cytometry analysis for measuring 5-FAM-rifampicin permeability into *M. tuberculosis* MLP cells. Density plots representing *M. tuberculosis* MLP cells without bead beating at: (**A**) 0 min, (**B**) 15 min, (**C**) 30 min, (**D**) 45 min and (**E**) 60 min of 5-FAM-rifampicin exposure. See the P2 gated population. (n = 3, indicated as MLP1, MLP2, MLP3)

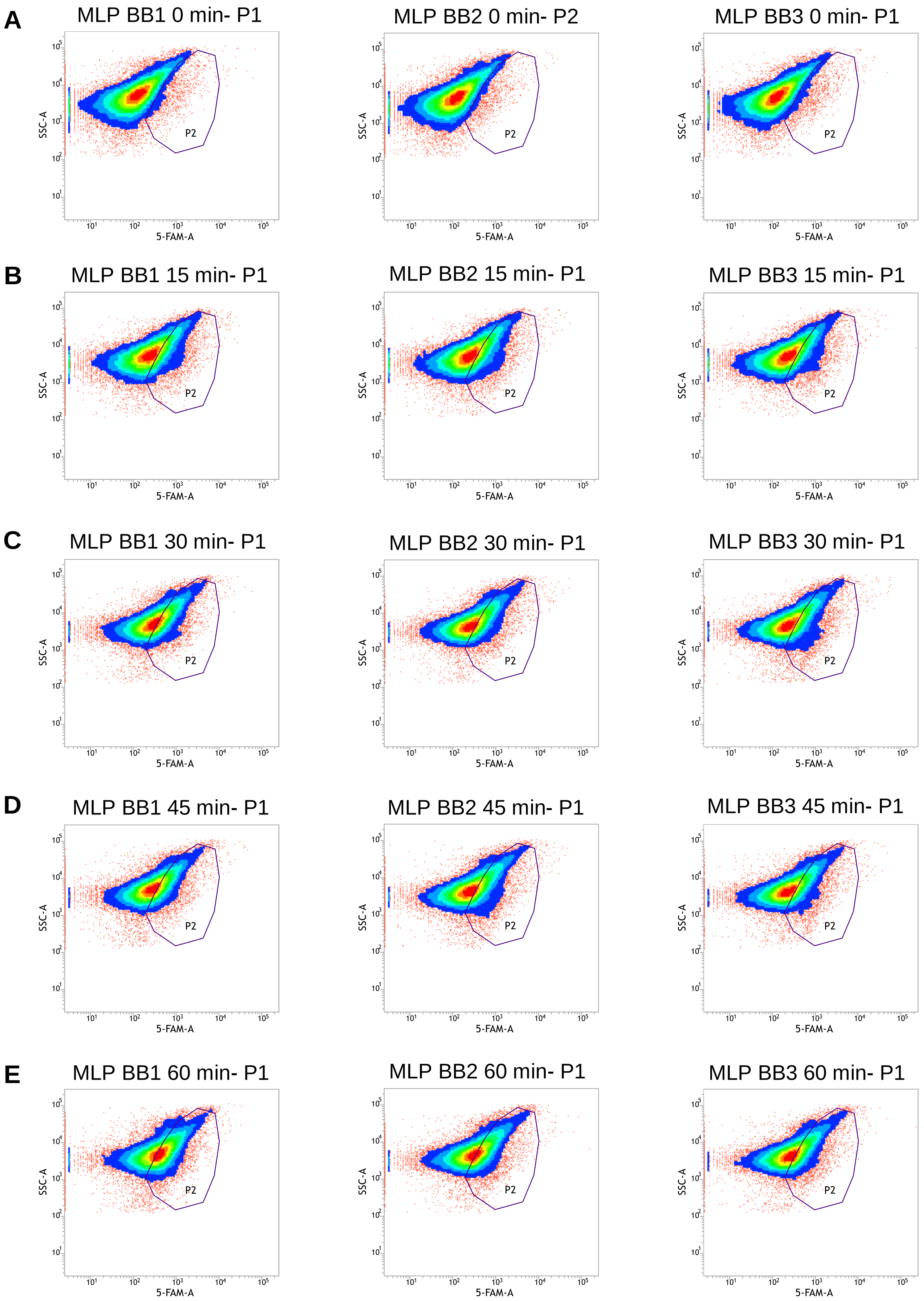

FIGURE S4. Flow cytometry analysis for measuring 5-FAM-rifampicin permeability into *M. tuberculosis* MLP bead-beaten cells. Density plots representing *M. tuberculosis* MLP bead-beaten (BB) cells at: (**A**) 0 min, (**B**) 15 min, (**C**) 30 min, (**D**) 45 min and (**E**) 60 min of 5-FAM-rifampicin exposure. See the P2 gated population. (n = 3 , indicated as MLP BB1, MLP BB2, MLP BB3)

A

MLP	Total No. of Cells	No. of cells in P2	Proportion of cells in P2 (%)	MLP BB	Total No. of Cells	No. of cells in P2	Proportion of cells in P2 (%)
**MLP1 0'**	28,580	321	1.12	**MLP BB1 0'**	32,048	1,646	5.14
**MLP2 0'**	28,430	367	1.29	**MLP BB2 0'**	32,005	1,506	4.71
**MLP3 0'**	28,219	299	1.06	**MLP BB3 0'**	32,017	1,816	5.67
**MLP1 15'**	28,046	3,961	14.12	**MLP BB1 15'**	30,819	7,376	23.93
**MLP2 15'**	28,150	4,125	14.65	**MLP BB2 15'**	30,567	8,057	26.36
**MLP3 15'**	28,250	3,540	12.53	**MLP BB3 15'**	30,140	6,719	22.29
**MLP1 30'**	28,717	7,755	27	**MLP BB1 30'**	29,655	11,359	38.3
**MLP2 30'**	28,974	9,587	33.09	**MLP BB2 30'**	29,561	10,098	34.16
**MLP3 30'**	29,359	5,825	19.84	**MLP BB3 30'**	30,260	9,514	31.44
**MLP1 45'**	29,198	13,336	45.67	**MLP BB1 45'**	29,728	10,380	34.92
**MLP2 45'**	29,052	11,573	39.84	**MLP BB2 45'**	30,060	10,329	34.36
**MLP3 45'**	30,680	10,439	34.03	**MLP BB3 45'**	29,590	8,955	30.26
**MLP1 60'**	29,436	12,883	43.77	**MLP BB1 60'**	30,137	15,610	51.8
**MLP2 60'**	28,776	15,158	52.68	**MLP BB2 60'**	29,608	13,096	44.23
**MLP3 60'**	29,619	14,009	47.3	**MLP BB3 60'**	29,609	11,193	37.8

B

RSP	Total No. of Cells	No. of cells in P2	Proportion of cells in P2 (%)	RSP BB	Total No. of Cells	No. of cells in P2	Proportion of cells in P2 (%)
**RSP1 0'**	41,000	1,104	2.69	**RSP BB1 0'**	34,385	4,733	13.76
**RSP2 0'**	38,070	1,109	2.91	**RSP BB2 0'**	32,477	4,465	13.75
**RSP3 0'**	43,518	2,465	5.66	**RSP BB3 0'**	33,009	5,160	15.63
**RSP1 15'**	39,520	3,413	8.64	**RSP BB1 15'**	35,187	9,197	26.14
**RSP2 15'**	38,396	1,837	4.78	**RSP BB2 15'**	34,834	10,011	28.74
**RSP3 15'**	40,917	5,456	13.33	**RSP BB3 15'**	34,068	8,175	24
**RSP1 30'**	39,744	3,635	9.15	**RSP BB1 30'**	37813	13,279	35.12
**RSP2 30'**	37,141	2,778	7.48	**RSP BB2 30'**	41397	15,557	37.58
**RSP3 30'**	38,204	5,456	14.28	**RSP BB3 30'**	37431	12,815	34.24
**RSP1 45'**	35,104	2,404	6.85	**RSP BB1 45'**	42,963	19,550	45.5
**RSP2 45'**	38,370	4,358	11.36	**RSP BB2 45'**	34,255	10,003	29.2
**RSP3 45'**	38,363	4,807	12.53	**RSP BB3 45'**	33,534	9,272	27.65
**RSP1 60'**	36,580	4,858	13.28	**RSP BB1 60'**	41,656	15,985	38.37
**RSP2 60'**	36,799	5,134	13.95	**RSP BB2 60'**	37,858	12,598	33.28
**RSP3 60'**	36,091	5,549	15.38	**RSP BB3 60'**	36,201	12,589	34.78

FIGURE S5. Quantitation of the cells in the P2 gate that gained 5-FAM-rifampicin fluorescence. (**A, B**) Tables showing the proportion of the cells of the MLP population and the rifampicin surviving population (RSP) in the P2 gate during the 60 min of 5-FAM-rifampicin exposure. The proportion and number of the cells in the P2 gate of: (**A**) MLP cells and (**B**) RSP cells. Statistical significance was calculated using paired *t* test (n = 3).

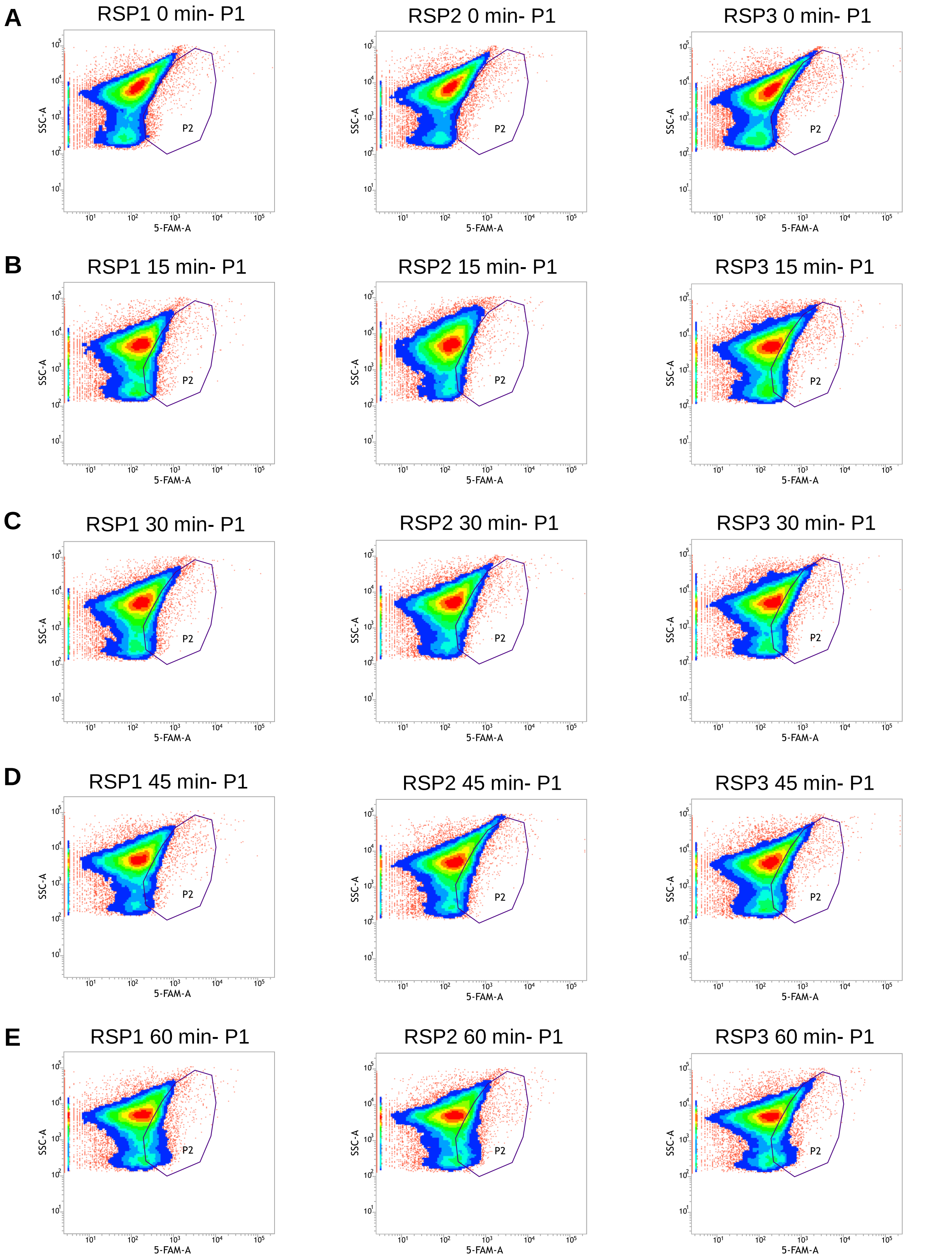

FIGURE S6. Flow cytometry analysis for measuring 5-FAM-rifampicin permeability into *M. tuberculosis* RSP cells. Density plots representing *M. tuberculosis* RSP cells without bead-beating at: (**A**) 0 min, (**B**) 15 min, (**C**) 30 min, (**D**) 45 min and (**E**) 60 min of 5-FAM-rifampicin exposure. See the P2 gated population. (n = 3 , indicated as RSP1, RSP2, RSP3)

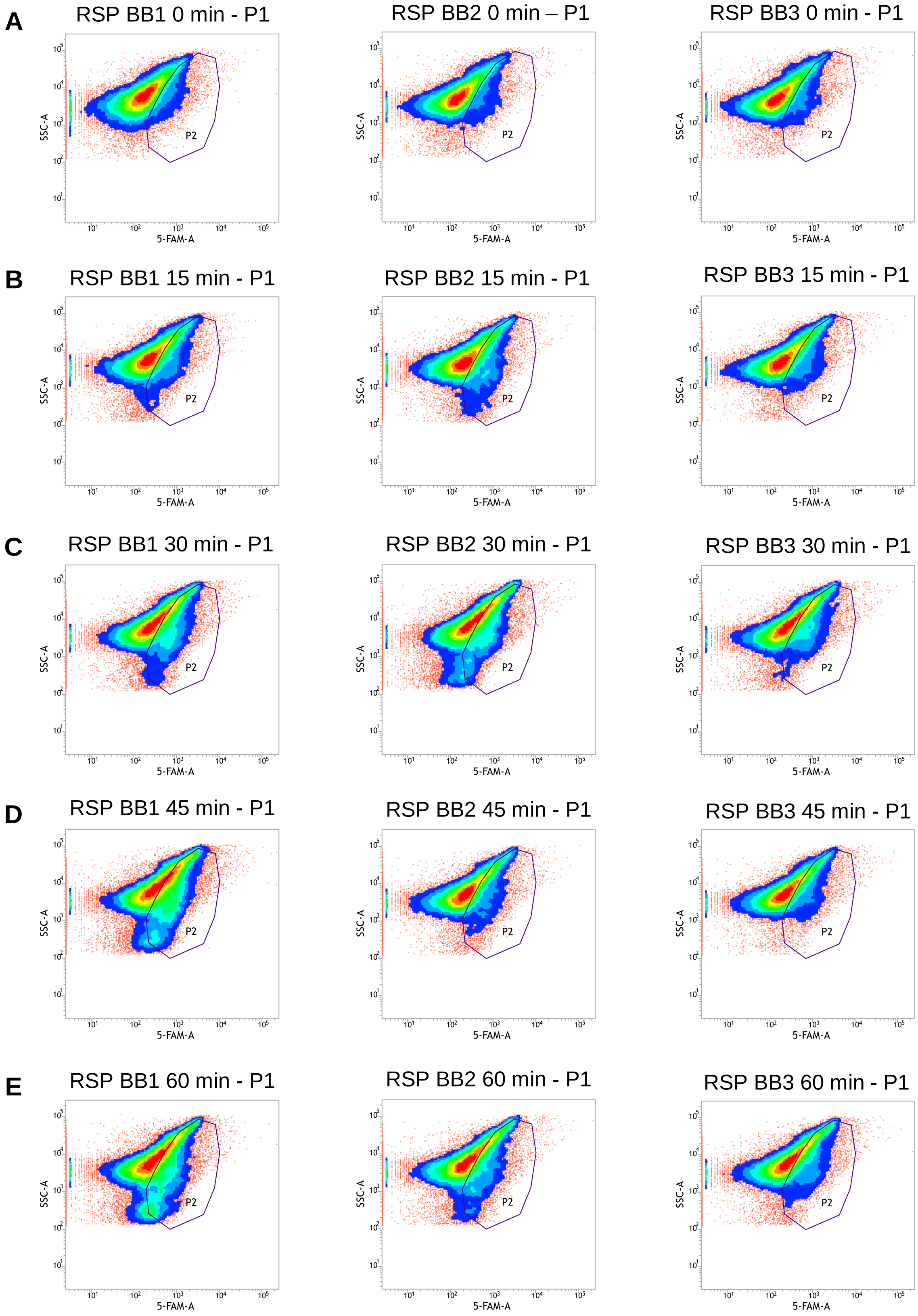

FIGURE S7. Flow cytometry analysis for measuring 5-FAM-rifampicin permeability into *M. tuberculosis* RSP bead-beaten cells. Density plots representing *M. tuberculosis* RSP bead-beaten (BB) cells at: (**A**) 0 min, (**B**) 15 min, (**C**) 30 min, (**D**) 45 min and (**E**) 60 min of 5-FAM-rifampicin exposure. See the P2 gated population. (n = 3 , indicated as RSP BB1, RSP BB2, RSP BB3)

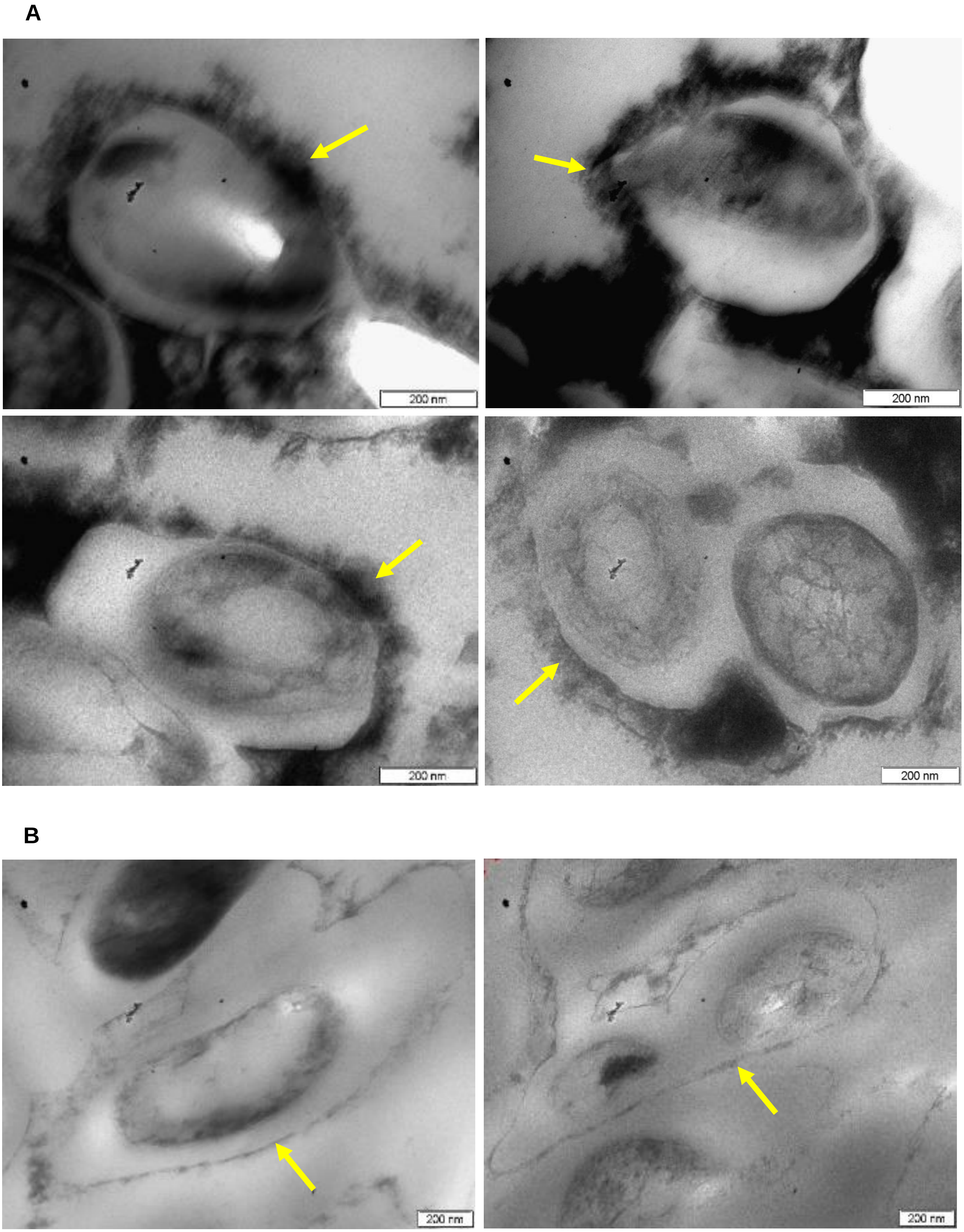

FIGURE S8. Transmission electron micrographs of *Mycobacterium tuberculosis* cells of the rifampicin surviving population (RSP) and MLP population with different capsular outer layer morphology. (**A**) RSP cells (12th day) with deeply stained TCOL. (**B**) MLP cells, cultured further for 12 days in the absence of rifampicin, with thin NCOL. Yellow arrows indicate TCOL/NCOL.

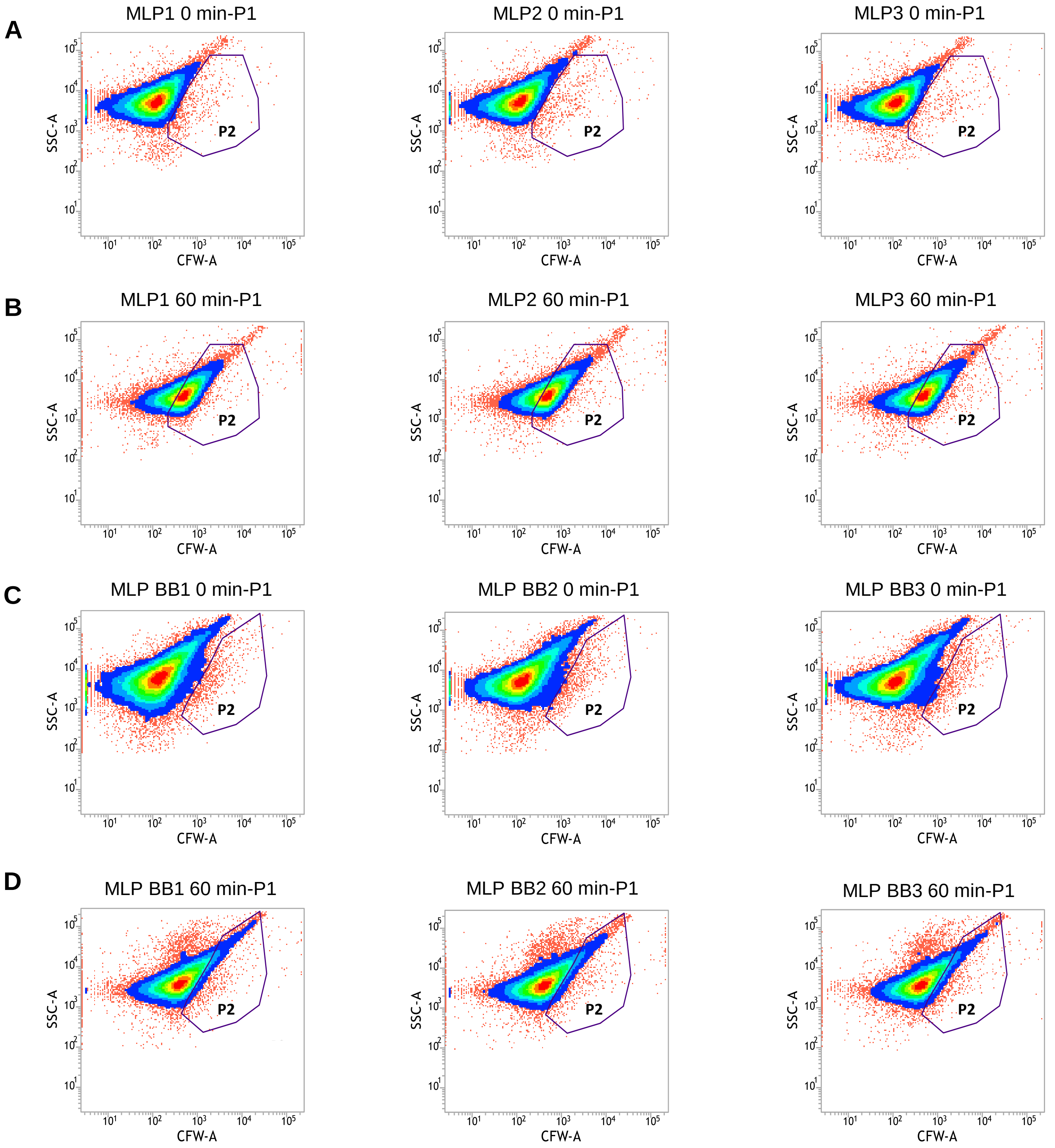

FIGURE S9. Flow cytometry profile of calcofluor white (CFW) stained *Mtb* MLP cells without and with bead beating. (**A, B**) MLP cells at 0 min and after 60 min of CFW staining, respectively. (**C, D**) MLP cells with bead beating at 0 min and after 60 min of CFW staining, respectively. See the P2 gated population that gained the stain during the 60 min. (n = 3). The flow cytometry data were analysed using FACSuite software. The P1 population was obtained by gating the whole population (all events) in each sample. The polygonal P2 gate was used to analyse the exact number of cells showing higher CFW fluorescence than the cells in P1 gate (polygonal P2 gate helped to exclude the cells in the P1 population). Further, the P2 gate was placed exactly where the P1 population ended in order to gate the actual number of cells showing higher CFW median fluorescence than the cells in the P1 gate.

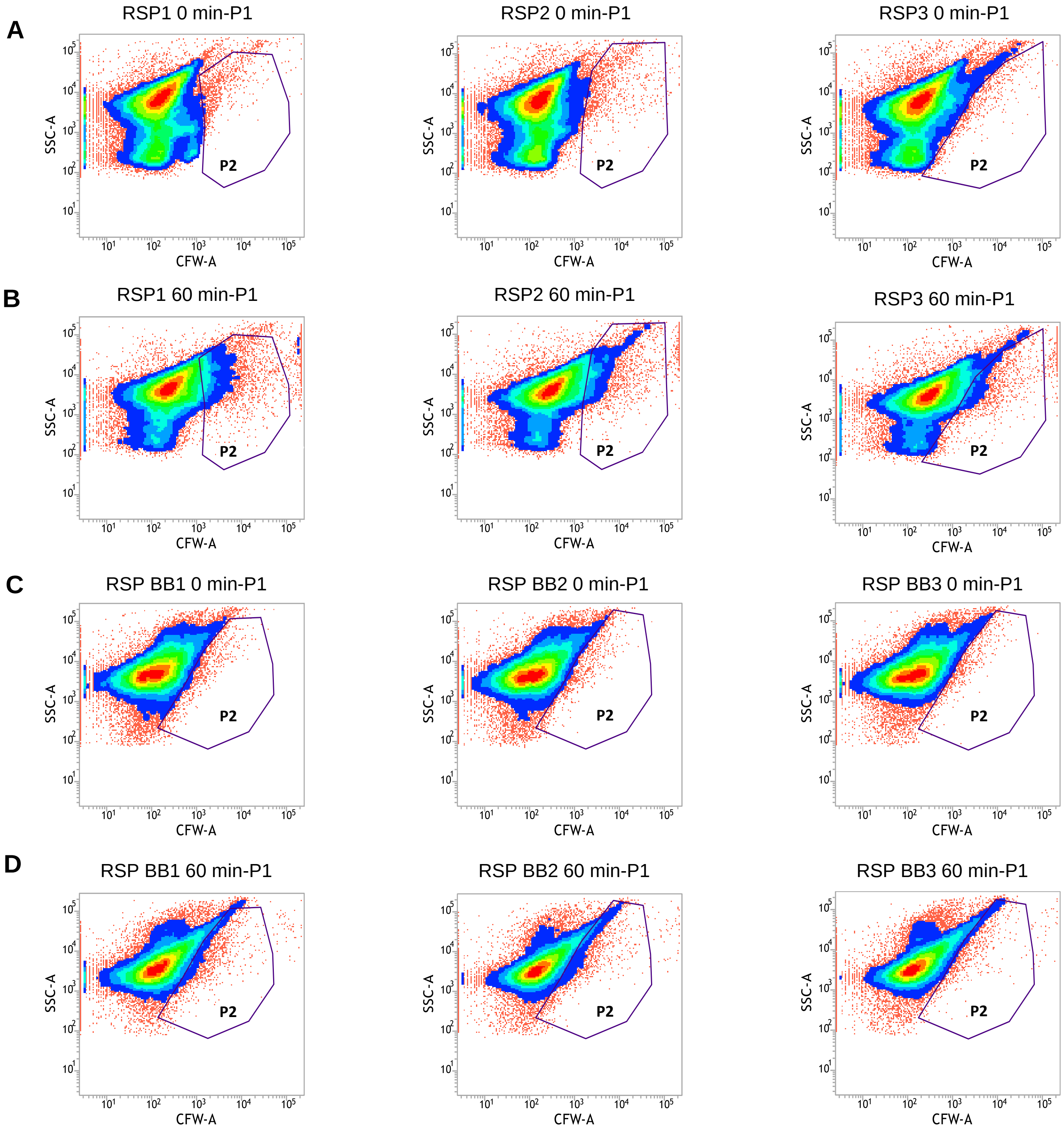

FIGURE S10. Flow cytometry profile of calcofluor white (CFW) stained *Mtb* RSP cells without and with bead beating. (**A, B**) RSP cells at 0 min and 60 min CFW staining duration, respectively. (**C, D**) RSP cells with bead beating at 0 min and 60 min CFW staining duration, respectively. See the P2 gated population that gained the stain during the 60 min. (n = 3). The flow cytometry data were analysed using FACSuite software. The P1 population was obtained by gating the whole population (all events) in each sample. The polygonal P2 gate was used to analyse the exact number of cells showing higher CFW fluorescence than the cells in P1 gate (polygonal P2 gate helped to exclude the cells in the P1 population). Further, the P2 gate was placed exactly where the P1 population ended in order to gate the actual number of cells showing higher CFW median fluorescence than the cells in the P1 gate.
